# Deregulated High Affinity Copper Transport Alters Iron Homeostasis in *Arabidopsis*

**DOI:** 10.3389/fpls.2020.01106

**Published:** 2020-07-23

**Authors:** Ana Perea-García, Amparo Andrés-Bordería, Francisco Vera-Sirera, Miguel Angel Pérez-Amador, Sergi Puig, Lola Peñarrubia

**Affiliations:** ^1^ Departamento de Biotecnología, Instituto de Agroquímica y Tecnología de Alimentos (IATA), Consejo Superior de Investigaciones Científicas (CSIC), Paterna, Valencia, Spain; ^2^ Departament de Bioquímica i Biologia Molecular and Estructura de Recerca Interdisciplinar en Biotecnologia i Biomedicina (ERI BIOTECMED), Universitat de València, Burjassot, Valencia, Spain; ^3^ Instituto de Biología Molecular y Celular de Plantas (IBMCP), Consejo Superior de Investigaciones Científicas (CSIC)—Universidad Politécnica de Valencia (UPV), Valencia, Spain

**Keywords:** *Arabidopsis thaliana*, copper uptake, high affinity copper importer 1, iron homeostasis, metal interactions, metal mobilization

## Abstract

The present work describes the effects on iron homeostasis when copper transport was deregulated in *Arabidopsis thaliana* by overexpressing high affinity copper transporters COPT1 and COPT3 (*COPT^OE^*). A genome-wide analysis conducted on *COPT1^OE^* plants, highlighted that iron homeostasis gene expression was affected under both copper deficiency and excess. Among the altered genes were those encoding the iron uptake machinery and their transcriptional regulators. Subsequently, *COPT^OE^* seedlings contained less iron and were more sensitive than controls to iron deficiency. The deregulation of copper (I) uptake hindered the transcriptional activation of the subgroup Ib of basic helix-loop-helix (*bHLH-Ib*) factors under copper deficiency. Oppositely, copper excess inhibited the expression of the master regulator *FIT* but activated *bHLH-Ib* expression in *COPT^OE^* plants, in both cases leading to the lack of an adequate iron uptake response. As copper increased in the media, iron (III) was accumulated in roots, and the ratio iron (III)/iron (II) was increased in *COPT^OE^* plants. Thus, iron (III) overloading in *COPT^OE^* roots inhibited local iron deficiency responses, aimed to metal uptake from soil, leading to a general lower iron content in the *COPT^OE^* seedlings. These results emphasized the importance of appropriate spatiotemporal copper uptake for iron homeostasis under non-optimal copper supply. The understanding of the role of copper uptake in iron metabolism could be applied for increasing crops resistance to iron deficiency.

## Introduction

Copper (Cu) and iron (Fe) are transition metals with redox properties that form coordination complexes with organic molecules, acting as essential cofactors in numerous proteins, including components of the respiratory and photosynthetic electron transport chains (Puig et al., 2007; Nouet et al., 2011; Yruela, 2013). However, these redox properties also make Cu and Fe potentially toxic since they facilitate the formation of reactive oxygen species (ROS). ROS produce damage at different levels, as they are able to react with proteins, DNA, and lipids in cell membranes, altering their function (Ravet and Pilon, 2013). Metal ion-dependent redox biology constitutes a fundamental theme of aerobic life. Nowadays, both multicopper oxidases (MCO), functioning as metallooxidases, and metalloreductases from the FERRIC REDUCTASE OXIDASE (FRO) family are necessary in redox cycling processes required for metal trafficking in eukaryotic cells (Kosman, 2018). Since evolved in an anaerobic environment, the proto-aerobe organisms developed metalloreductases to supply reduced metals Fe^2+^ and Cu^+^ to transporters. Changes in the bioavailability of both metals throughout the evolution of the atmosphere led to a decrease and increase in the Fe and Cu bioavailability, respectively, allowing their substitution as cofactors in different proteins to perform similar functions (Crichton and Pierre, 2001). For instance, in *Arabidopsis thaliana*, Cu/Zinc (Zn) superoxide dismutase (SOD) is replaced by its Fe counterpart when Cu is scarce (Yamasaki et al., 2007). Metalloprotein substitution contributes to the increase of Fe content under Cu deficiency and vice versa (Waters et al., 2012).

Higher plants have developed sophisticated mechanisms to efficiently acquire and use micronutrients such as Cu and Fe. Cu and specially Fe deficiencies cause losses in agriculture by decreasing the productivity and nutritional value of crops. The deficiency of Fe in agriculture is due to its low bioavailability, especially in alkaline soils (Marschner, 2012). From the two classically described pathways for Fe acquisition in plants, *Arabidopsis* uses strategy I that is based on the reduction of Fe^3+^ to Fe^2+^ by the reductase FRO2 present in the root plasma membrane (Robinson et al., 1999). The ZIP-type divalent cation transporter IRON-REGULATED TRANSPORTER 1 (IRT1) incorporates the Fe^2+^ into the root cell (Connolly et al., 2002; Varotto et al., 2002; Vert et al., 2002). In the chloroplast, Fe participates in the electron transport chain, chlorophyll biosynthesis, the assembly of Fe/S clusters, and the biosynthesis of heme groups, among other processes (Yruela, 2013). Mitochondria are also high Fe consumer organelles mainly for the electron transport chain and the assembly of Fe/S groups (Nouet et al., 2011). Therefore, due to the possibility of forming ROS, Fe is complexed by the ferritin protein (Reyt et al., 2015). The transcription factor FIT (bHLH29) (FER-like IRON DEFICIENCY INDUCED TRANSCRIPTION FACTOR) is a basic helix-loop-helix (bHLH) that binds to DNA in response of Fe deficiency (Colangelo and Guerinot, 2004). FIT interacts and forms heterodimers with the subgroup Ib of bHLH proteins (bHLH38, bHLH39, bHLH100 and bHLH101), which are required to properly respond to Fe deficiency (Wang et al., 2013). Among the genes activated by FIT are those encoding the reductase *FRO2* and the Fe and Cu transporters *IRT1* and *COPT2*, respectively (Colangelo and Guerinot, 2004). FIT activity is regulated at multiple levels by hormones, oxidative stress, and other signals, being considered as a hub modulating the *Arabidopsis* strategy I response to Fe deficiency (Kobayashi, 2019).

Other regulators of Fe uptake are the IRON MAN/FE-UPTAKE-INDUCING PEPTIDE (IMA/FEP), a group of conserved plant peptides induced under Fe deficiency in vascular tissues (Grillet et al., 2018; Hirayama et al., 2018). Moreover, several phytohormones are involved in modulating Fe deficiency responses in plants. Ethylene, auxin, gibberellin, and salicylic acid function as positive factors of the Fe-deficiency responses, whereas cytokinin, brassinosteroid, abscisic acid, and jasmonic acid act as negative factors of Fe uptake (Kobayashi, 2019). The balance between cellular proliferation and differentiation in the *Arabidopsis* root has been attributed to the transcription factor UPBEAT1 (UPB1), which regulates this balance through ROS control (Tsukagoshi et al., 2010). Among the upstream regulators is ILR3 (bHLH105), from the subgroup IVc of bHLH transcription factors, which is induced under Fe deficiency and inhibits the expression of genes such as *At-NEET*, *FER1*, and *FER3* (Tissot et al., 2019). ILR3 and bHLH115 interact with the E3 ubiquitin ligase BRUTUS (BTS), which ubiquitinates them for degradation (Selote et al., 2015). BTS is a protein that participate in Fe sensing, with highly conserved domains including 3 hemerythrin domains, and a protein-protein interaction domain denoted REALLY INTERESTING NEW GENE (RING) (Long et al., 2010; Kobayashi et al., 2013). BTS and its paralogs are negative regulators of Fe assimilatory responses (Hindt et al., 2017).

Cu is found in the form of Cu^2+^ in the soil, although under metal deficiency it is introduced in the root cells as Cu^+^ by high affinity Ctr transporters, denoted COPT (COPPER TRANSPORTERS) in plants (Puig, 2014; Peñarrubia et al., 2015). Previous to Cu^+^ uptake, reductases, from the FRO family participate in the reduction of Cu^2+^ (Bernal et al., 2012). The Ctr protein family is conserved in eukaryotes and mediates cellular Cu^+^ acquisition (Nevitt et al., 2012). The recently solved X-ray structure of a Ctr member has confirmed that each monomer contains three transmembrane segments that assemble as homotrimers or heterotrimers and that Cu^+^ is incorporated through a central pore (Ren et al., 2019). From the six genes identified in *Arabidopsis* that encode COPT transporters (Sancenón et al., 2003; Garcia-Molina et al., 2013), *COPT1* and *COPT3* are both induced by Cu deficiency and their encoded proteins are located into plasma membrane and at a compartment of the secretory pathway, respectively (Andrés-Colás et al., 2010; Andrés-Colás et al., 2018). The phenotypes of plants with altered levels of COPT1 and COPT3 correlate with their expression at the pollen grains and suggest a predominant role of COPT1, also expressed at the root tip, in the acquisition of Cu from the soil (Sancenón et al., 2004; Andrés-Colás et al., 2010; Andrés-Colás et al., 2018). Constitutive *COPT1* and *COPT3* overexpression (*COPT^OE^* plants) lead to increased Cu uptake, oxidative stress, and phenotypes related to altered circadian rhythms (Andrés-Colás et al., 2010; Rodrigo-Moreno et al., 2013; Perea-García et al., 2016; Sanz et al., 2019). Furthermore, the *Arabidopsis* COPT1 transporter has been overexpressed in *Oryza sativa* plants, leading to increased Fe content in polished grains (Andrés-Bordería et al., 2017). These results underscore the effects of Cu status on Fe traffic and mobilization. Moreover, COPT2 is the *Arabidopsis* transporter with the highest expression along the root, and plants defective in *COPT2* are more resistant to double Cu and Fe deficiency (Perea-García et al., 2013). In fact, the *COPT2* promoter contains E-Box elements to which FIT binds (Colangelo and Guerinot, 2004). On the other hand, Cu deficiency involves the regulation of many metabolic processes to allocate the little Cu present to essential proteins. The Zn finger transcription factor SQUAMOSA-PROMOTER BINDING-LIKE PROTEIN 7 (SPL7) binds to GTAC consensus sequences present in the promoters of various genes that are expressed under Cu deficiency, such as *COPT2* (Yamasaki et al., 2009; Bernal et al., 2012). The presence of both cis regulatory elements justifies *COPT2* induction in the double deficiency of Cu and Fe (Perea-García et al., 2013).

The importance of the regulation of metal homeostasis, at both the cellular and the systemic levels, and its implications in agriculture and human health is evident. However, the molecular mechanisms underlying the interaction between both metals remain poorly understood (Gulec and Collins, 2014). As far as metal homeostasis networks for a specific metal are becoming well understood, the crosstalk between different metals is starting to emerge as a possibility to improve global metal nutritional levels for optimal organismal performing.

## Materials and Methods

### Plant Growth Conditions and Treatments

Seeds of *A. thaliana*, ecotype *Columbia-0* (Col-0) and of the transgenic lines *COPT1^OE^* and *COPT3^OE^* were surface-sterilized and stratified for 2 days at 4**°**C and were germinated in ½ MS medium (Sigma) plates including 1% sucrose (Murashige and Skoog, 1962) (½ MS) or supplemented with 10 μM CuSO_4_ (½ MS + 10 Cu) for the microarray analysis. Seedlings were grown as previously described (Andrés-Colás et al., 2010) for 7 days with a 12 h neutral photoperiod (65 μmol m^−2^ of cool-white fluorescent light) at 23**°**C/16**°**C temperature cycle.

In order to obtain ½ MS medium with the indicated concentrations of either Cu or Fe, the solution was prepared by adding macronutrients (Sigma) and micronutrients consisted in a mix of 50 μM H_3_BO_3_, 36.6 μM MnSO_4_ H_2_O, 15 μM ZnSO_4_ 7H_2_O, 0.57 μM NaMoO_4_ 2H_2_O, 0.25 mM KI, and 0.05 μM CoCl_2_ 6H_2_O. Finally, 0.05% MES, 1% sucrose, and 0.8% phytoagar was added, and the pH was adjusted to 5.7–5.8 with diluted KOH. The Cu concentration in the Cu deficiency (0 μM CuSO_4_) media including commercial phytoagar (Duchefa Biochemie) measured by ICP-MS is <0.008 μM Cu. To study the effects that the different Cu and Fe content have in plants, 50 μM Fe-citrate and 1 μM CuSO_4_ 5H_2_O were added to the medium for Cu and Fe sufficiency conditions. Moreover, seedlings were grown in Cu deficiency (0 μM CuSO_4_) and Cu excess (10 μM CuSO_4_). On the other hand, Fe-sufficient and slight or severe Fe deficiency medium was supplemented with 50, 10 and 0 μM Fe-citrate, respectively. Other metal concentrations and treatments were used for specific experiments as indicated in the Supplementary Figure Legends.

The chlorophyll content in seedlings and leaves was determined by the trichlorometric method (Parsons and Strickland, 1965). Root length was measured using the Image J 1.42q software (http://rsb.info.nih.gov./ij). Values represent the arithmetic mean ± standard deviation (SD) of three biological replicates (n = 3).

For the determination of ferroreductase activity, three seedlings of 7-day-old were collected and weighed. Next, a 1:1 mixture made with 300 μM bathophenantroline disulfonate (BPDS, Sigma) and 100 μM Fe III-EDTA was added to the seedlings and incubated at 30°C with stirring 225 rpm in the dark. After 30 min, the solution was collected and absorbance A_535_ was measured in a spectrophotometer (Grillet et al., 2014). Values represent the arithmetic mean ± standard deviation (SD) of three biological replicates (n = 3).

For root Fe^3+^ detection by Perl’s staining, four to five seedlings of 11-day-old were vacuum infiltrated with equal volumes of 4% (v/v) HCl and 4% (w/v) K- ferrocyanide (Perl´s stain solution for 15 min) and incubated at room temperature for 30 min (Stacey et al., 2008). One representative photograph is shown in the figure. This method is based on the Fe^3+^ dependent conversion of ferrocyanide into insoluble crystals of Prussian blue under acidic conditions. Localization of Fe^3+^ was observed and analyzed with a (Olympus CX41) microscope equipped with (Leyca MC170HD) camera and (LAS V4.10) software.

### Microarrays and Bioinformatics

Seven-day-old seedlings of the WT and the *COPT1^OE^* line were grown in the 12 h neutral photoperiod and three biological replicates were obtained for the (½ MS) treatment and four biological replicates were used as 10 μM CuSO_4_, (½ MS + 10 Cu) samples. Total RNA was isolated using the RNeasy Plant Mini Kit (Qiagen) and aRNA was amplified using the MessageAmp™ II aRNA Amplification kit (Ambion). Long oligonucleotide microarrays were provided by Dr. David Galbraith (University of Arizona, http://www.ag.arizona.edu/microarray/). The hybridization and analysis were performed as described elsewhere (Bueso et al., 2007). The expression values (log_2_) were obtained using the GenePix Pro 6.0 microarray-analysis software (Molecular Devices, Sunnyvale CA) and normalized with the GenePix Pro 6.0 and Acuity 4.0 software (Molecular Devices, Sunnyvale CA). Differential genes were identified with significance analysis of microarray (SAM) (Tusher et al., 2001) with false discovery rate (FDR) of <6% and 2-fold change (log_2_ ≤|1|). Biological processes were identified with the Gene Ontology (GO) annotation (Ashburner et al., 2000), performed by the GeneCodis2.0 (http://genecodis.dacya.ucm.es/) (Carmona-Saez et al., 2007; Nogales-Cadenas et al., 2009) program (**Table 1**). The total differentially regulated genes are shown as Supplementary material (**Supplementary Tables SI** and **SII**). The microarray raw data were deposited in the NCBI’s Gene Expression Omnibus (Edgar, 2002) and are accessible through GEO Series accession number GSE143857.

**Table 1:** Differentially expressed iron- and tetrapyrrole-related genes in *COPT1^OE^* vs. WT seedlings in low and high Cu.

Gene Name	ID Gen	Description	Value	
			MS	Cu
**Fe homeostasis**				
*IRT1*	AT4G19690	*Iron-regulated transporter 1*	**−1.74**	**−1.77**
*FRO2*	AT1G01580	*Ferric reduction oxidase 2*	---	**−1.30**
*FRO3*	AT1G23020	*Ferric reduction oxidase 3*	0.01	**1.24**
*COPT2*	AT3G46900	*Copper transporter 2*	**−0.92**	−0.05
*CYP82C4*	AT4G31940	*Cytochrome P450*	–	**−2.59**
*FER1*	AT5G01600	*Ferritin 1*	−0.15	**−2.17**
*FER3*	AT3G56090	*Ferritin 3*	−0,72	−0.71
*At-NEET*	AT5G51720	*Fe metabolism*	−0.51	**−2.30**
*NAS4*	AT1G56430	*Nicotianamine syntase 4*	**−1.51**	0.64
*OPT3*	AT4G16370	*Oligopeptide transporter 3*	**−1.14**	0.79
**Fe regulators**				
*FIT/bHLH29*	AT2G28160	*BHLH 29*	–	−0.39
*bHLH039*	AT3G56980	*Basic helix-loop-helix protein 39*	−0.15	**2.52**
*bHLH101*	AT5G04150	*Basic helix-loop-helix protein 101*	0.87	**2.15**
*bHLH100*	AT2G41240	*Basic helix-loop-helix protein 100*	0.22	**2,94**
*bHLH115*	AT1G51070	*Basic helix-loop-helix protein 115*	**−1.14**	−0.05
*UPB1*	AT2G47270	*Basic helix-loop-helix UPBEAT1*	**1.79**	−0.06
*BTS*	AT3G18290	*BRUTUS RING-ubiquitin ligase*	−0.26	**1.36**
*FEP2/IMA2*	AT1G47395	*Fe-Uptake-Inducing peptide2*	0.29	**3.32**
*FEP3/IMA1*	AT1G47400	*Fe-Uptake-Inducing peptide3*	0.42	**2.47**
***Tetrapyrrole retrograde signaling***				
*CA1*	AT3G01500		−0.32	**−1.06**
*LHCB1.1*	AT1G29920		0.34	**−0.97**
*LHCB1.4*	AT2G34430		–	**−1.10**
*LHCB2.3*	AT3G27690		–	**−1.80**
*LHCB4.1*	AT5G01530		–	**−1.30**
*LHCB4.3*	AT2G40100		–	**−1.34**
*LHCB5*	AT4G10340		**−1.12**	**−0.73**
***Components***				
*HEMA1*	AT1G58290	*glutamyl-tRNA reductase 1, chloroplast*	−2.13	**−1.46**
*GUN4*	AT3G59400	*genome uncoupled 4*	−1.00	**−1.36**
*CHL27*	AT3G56940	*Copper response defect 1*	−0.10	−0.76
*SIG1*	AT1G64860	*Chloroplast sigma factor 1*	−1.70	−1.16

Gene name, MIPS code, gene description, and the expression values (fold change) of differentially regulated genes with a log_2_ ratio of ≥|1| in the COPT1^OE^ versus the WT were indicated under –Cu (MS) and + 10 μM Cu (Cu). In bold are the genes that passed the statistical analysis.

### Gene Expression by Real-Time Quantitative PCR

Total *Arabidopsis* RNA was isolated using the RNeasy Plant Mini Kit (Qiagen), was quantified by UV spectrophotometry and its integrity was visually assessed on ethidium bromide-stained agarose gels. After treatment with Dnase I Amp Grade (Invitrogen), cDNA was generated by retro-transcriptase SSII (Invitrogen) as previously described (Andrés-Colás et al., 2006). Real-time quantitative PCR (RT-qPCR) was carried out with SYBR-Green qPCR Super-Mix-UDG with ROX (Invitrogen) with the specific primers detailed in **Table SVI** in a CFX96 Touch™ Real Time PCR Detection System (BioRad), with one cycle of 95°C for 2 min and 40 cycles consisting in 95°C for 30 s and 60°C for 30 s. Expression values were normalized to *UBQ10* and to the WT in deficiency conditions using the 2^−ΔΔ^ ^Ct^ method. Values represent the arithmetic mean ± standard deviation (SD) of three biological replicates (n = 3).

### Oxygen Consumption Determination

To study O_2_ consumption, 14–16 roots of 10-day-old seedlings, grown in different conditions, were used. The roots were cut with a scalpel and resuspended in 1.5 ml of ½ MS liquid medium, as it is described above but without sucrose. The roots were transferred to an airtight chamber and the measurement of O_2_ consumption for a minimum of 5 min was performed using a Clark type electrode (Oxyview system). The rate of decrease of O_2_, referenced to fresh weight (F.W.) of the roots (nmol O_2_/OD_600_ × F.W.) was taken as an index of respiratory capacity. Values represent the arithmetic mean ± standard deviation (SD) of three biological replicates (n = 3).

### Metal and Hormone Determinations

Cu and Fe contents were determined by ICP-MS as described previously (Andrés-Colás et al., 2006; Carrió-Seguí et al., 2015) at the Servei Central de Suport a la Investigació Experimental (SCSIE) of the Universitat de València.

For the determination of the content of ABA, indol-3-acetic acid (IAA), and JA, 8-day-old seedlings were lyophilized, processed and analyzed by UHPLC (ultra-high-pressure liquid chromatography) Q-Exactive (ThermoFisher Scientific) as described previously (da Silva et al., 2017) in the plant hormone quantification service of the Institute of Molecular and Cellular Plant Biology (IBMCP, Valencia). Values represent the arithmetic mean ± standard deviation (SD) of three biological replicates (n = 3).

### Statistical Analysis

The statistical analysis of the relative gene expression was performed by the pair wise fixed reallocation randomization test (*p*-value <0.05) (Pfaffl, 2002). For the remaining parameters, the analysis was carried out using one or two-way ANOVA with the means compared by the Duncan test or a Kruskal–Wallis (*p*-value <0.05) test for a non-parametric measurements using the InfoStat software, version 2010 (http://www.infostat.com.ar) (Di Rienzo et al., 2011).

## Results

### A Genome-Wide Expression Analysis Highlights That Iron Homeostasis Is Affected in *COPT1^OE^* *Arabidopsis* Plants

To identify at a molecular level the global effects caused by the deregulation of Cu homeostasis in *Arabidopsis*, we performed a comparative transcriptomic analysis of 7-day-old wild-type (WT) and a previously generated COPT1 overexpressing (*COPT1^OE^*) line (Andrés-Colás et al., 2010), grown under Cu deficiency (½ MS) and mild Cu excess (½ MS + 10 Cu) conditions. The induction of *COPT1* expression under Cu deficiency in the WT was corroborated by RT-qPCR, as well as its overexpression in *COPT1^OE^* seedlings, both under deficiency and excess Cu conditions, in the samples that were used in the hybridizations of the DNA microarrays (data not shown). From the global analysis of gene expression, a total of 583 differentially expressed genes were identified with a log_2_ ratio of ≥|1| in the *COPT1^OE^* versus WT (**Supplementary Figure S1** and **Tables SI** and **SII**). These were distributed in a total of 482 induced (ratio ≥1) and of 101 repressed genes (ratio ≥−1) in *COPT1^OE^* seedlings for the two growth conditions tested (**Supplementary Figure S1** and **Supplementary Tables SI** and **SII**).

The number of genes induced was greater than that of genes repressed in the two conditions. On the other hand, the mild Cu excess condition showed the greatest number of genes with differential expression compared to Cu deficiency, with 393 genes (312 induced and 81 repressed) compared to 160 genes (142 induced and 18 repressed), respectively. The analysis of the gene ontology (GO) of differentially regulated genes between WT and *COPT1^OE^* seedlings indicated that several the Biological Processes categories were overrepresented, including those linked to photosynthesis, photomorphogenesis, transport of metals such as Fe, Zn, manganese, cadmium, and phosphate, oxidative stress and responses to abscisic acid (ABA) and cold stress (**Supplementary Table SIII**). On the other hand, the Molecular Function of genes differentially regulated in *COPT1^OE^* included the chlorophyll and tetrapyrrole binding (**Supplementary Table SIV**). GO analysis also revealed that the chloroplast was the subcellular compartment most affected by the changes observed in *COPT1^OE^*, as 39% of the significantly enriched GO categories of Cellular Compartment are related to chloroplast (chloroplast, chloroplast envelope, thylakoid membrane, chloroplast membrane, stroma, photosystems I and II, and light-harvesting complex) (**Supplementary Table SV**). The Fe-related genes studied in this work, which expression was affected in *COPT1^OE^* plants, are summarized in **Table 1**.

### Fe Assimilation in the *COPT^OE^* Seedlings

**Figure 1:**
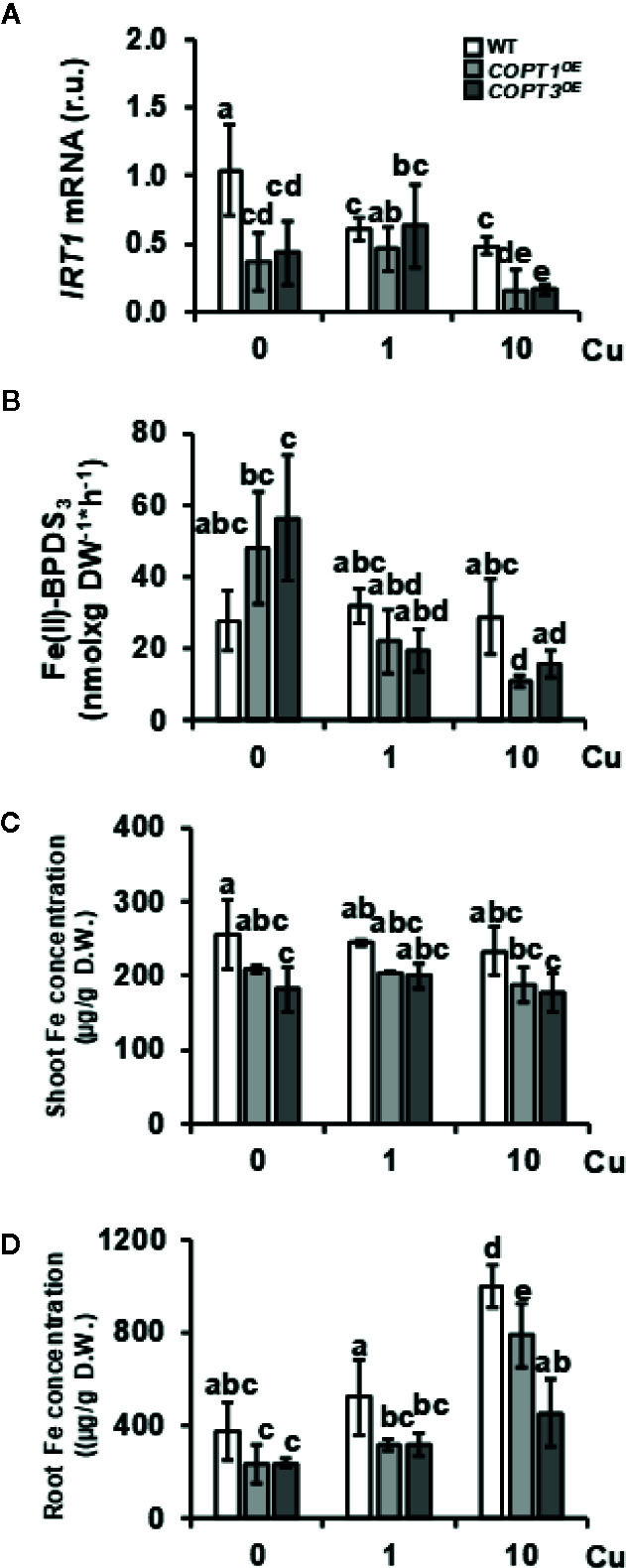
Expression of strategy I components of Fe uptake in *COPT^OE^* seedlings. **(A)** Relative expression of *IRT1* gene in 7-day-old WT (white bars), *COPT1^OE^* (light grey bars), and *COPT3^OE^* (dark grey bars) seedlings grown under Cu deficiency (0 μM CuSO_4_), sufficiency (1 μM CuSO_4_) or Cu excess (10 μM CuSO_4_) was determined by RT-qPCR. Gene expression is represented as relative expression levels (r.u.) in relation to the WT under control conditions. *UBQ10* was used as housekeeping gene. Bars correspond to arithmetic means (2^−ΔΔCt^) ± SD of biological replicates (n = 3). **(B)** Ferroreductase activity in 7-day-old WT and *COPT^OE^* seedlings grown in the same conditions as in **(A)** measured at A_535_ nm. Bars correspond to arithmetic means ± SD of biological replicates (n = 3) **(C)** Shoot and **(D)** root Fe concentration in 7-day-old WT, *COPT1^OE^* and *COPT3^OE^* seedlings grown on CuSO_4_ media specified in **(A)** and determined as μg/g of dry weight (D.W.). Bars correspond to arithmetic means ± SD of biological replicates (n = 3). Samples with a letter in common are not significantly different (*p*-value <0.05). D.W., dry weight.

One of the most relevant result of the transcriptomic analysis in *COPT1^OE^* is the altered expression of Fe-related genes (**Table 1**), such as *IRT1* and *FRO*2, involved in the strategy I of Fe uptake (**Table 1**). Expression of the Fe transporter *IRT1* was reduced in *COPT1^OE^* seedling in both Cu deficiency (½ MS) or excess (½ MS + 10 Cu). In contrast, the genes encoding *FRO* reductases showed a different behavior; expression of *FRO2* was induced but that for *FRO3* was repressed in *COPT1^OE^* seedlings only in Cu excess. In order to further assess the effects of Cu on their expression, seedlings were grown on hand-made ½ MS medium with three different CuSO_4_ concentrations, including Cu deficiency (0 μM CuSO_4_), sufficiency (1 μM CuSO_4_), and mild Cu excess (10 μM CuSO_4_). Furthermore, in addition to *COPT1^OE^*, plants overexpressing the *COPT3* transporter (*COPT3^OE^*) (Andrés-Colás et al., 2010) were also included to further assess the effect of deregulated Cu^+^ entrance (**Figure 1**). *IRT1* expression slightly decreased in the WT seedlings as Cu increased in the medium, being significantly lower under Cu sufficiency and excess compared to deficiency conditions (**Figure 1A**). Similar to the global transcriptomic analysis, *IRT1* expression in *COPT^OE^* (*COPT1^OE^* and *COPT3^OE^* lines) was lower than in WT under both Cu deficiency and excess. Since genes encoding reductases *FRO2* and *FRO3* displayed distinct regulation in *COPT1^OE^* seedlings (**Table 1**), total ferroreductase activity was measured in the roots of 7-day-old *COPT^OE^* seedlings (**Figure 1B**). No changes in ferroreductase activity were observed under Cu deficiency and sufficiency. However, a slightly lower ferroreductase activity was observed under Cu excess in the *COPT1^OE^* plants compared to the WT (**Figure 1B**).

*COPT1^OE^* and *COPT3^OE^* seedlings were previously shown to incorporate more Cu, both under metal deficiency and excess in the growth medium (Andrés-Colás et al., 2010). Now, the Fe content was determined by ICP-MS in both shoots and roots from 7-day-old WT and *COPT^OE^* seedlings grown under the different Cu conditions (**Figures 1C, D**). We observed that *COPT^OE^* seedlings had slightly lower endogenous Fe concentration than the WT both in shoots and roots but only significant under Cu sufficiency and excess. This decrease was more evident in the roots of the *COPT3^OE^* seedlings (**Figure 1D**), which is consistent with their exacerbated reduction in *IRT1* expression (**Figure 1A**).

**Figure 2:**
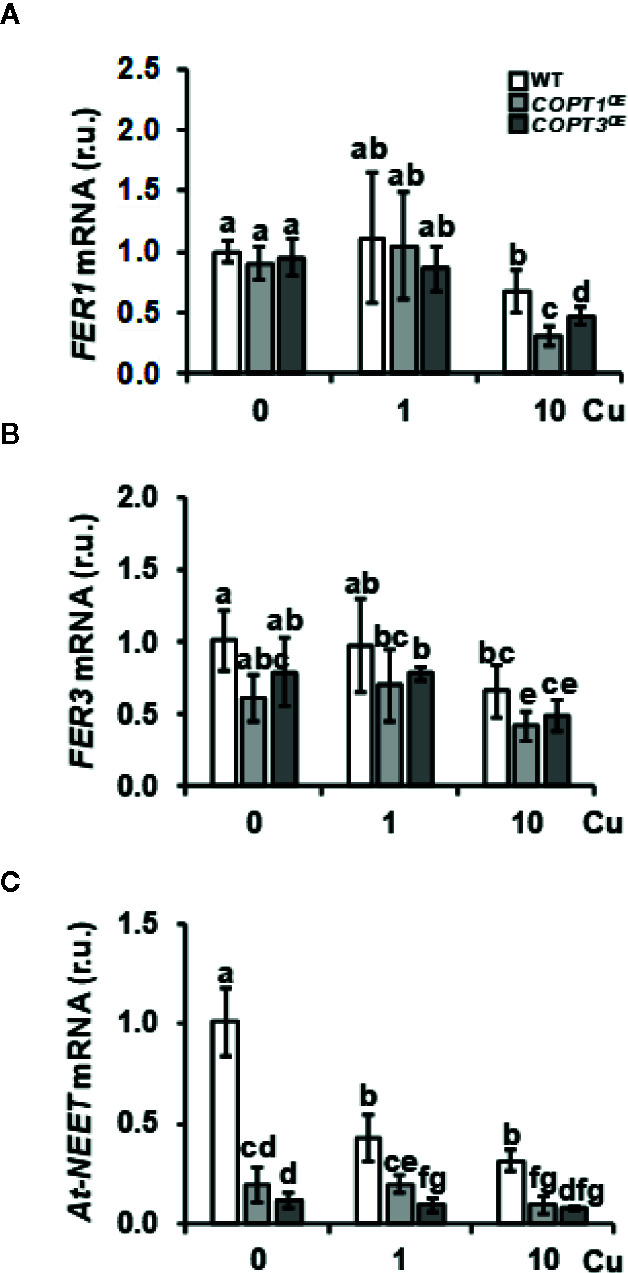
Expression of Fe metabolism genes in *COPT^OE^* seedlings. Relative expression of *FER1* **(A)**, *FER3* **(B)**, and *At-NEET* **(C)** genes in WT (white bars), *COPT1^OE^* (light grey bars), and *COPT3^OE^* (dark grey bars) seedlings grown under Cu deficiency (0 μM CuSO_4_), sufficiency (1 μM CuSO_4_) or Cu excess (10 μM CuSO_4_) was determined by RT-qPCR. Gene expression is represented as relative expression levels (r.u.) in relation to the WT under control conditions. *UBQ10* was used as housekeeping gene. Bars correspond to arithmetic means (2^−ΔΔCt^) ± SD of biological replicates (n = 3). Samples with a letter in common are not significantly different (*p*-value <0.05).

To corroborate the expression changes of other Fe-related genes involved in Fe storage and metabolism in *COPT1^OE^* seedlings, we further investigated the expression of genes included in these processes, such as *FER1*, *FER3*, and *At-NEET* (**Table 1** and **Figure 2**). Previous data have shown that the expression of *FER1* is higher among the four genes (*FER1*, *FER2*, *FER3* and *FER4*) that encode ferritins (Petit et al., 2001). *FER1* and *FER3* expression was down-regulated under Cu excess compared to Cu deficiency in WT and this decrease was more exacerbated in *COPT^OE^* seedlings (**Table 1** and **Figures 2A, B**). This pattern is also in agreement with the lower Fe content in the *COPT^OE^* under Cu excess (**Figure 1C**). *At-NEET* encodes a Fe/S protein involved, among other processes, in the Fe metabolism and ROS homeostasis (Nechushtai et al., 2012; Mittler et al., 2019). *At-NEET* expression was induced under Cu deficiency in the WT and reduced in the *COPT^OE^* seedlings under all the Cu conditions studied (**Table 1** and **Figure 2C**). The expression of *FER1*, *FER3*, and *At-NEET* was also analyzed under different Cu and Fe contents in WT seedlings (**Supplementary Figure S2**). Whereas all of them were induced under Cu deficiency compared to control conditions, only *FER1* expression was repressed under Fe deficiency. *FER3* and *At-NEET* were not regulated by Fe levels (**Supplementary Figure S2**). Altogether, these results indicate that the deregulated Cu^+^ entrance in *COPT^OE^* prevents the induction of genes involved in Fe uptake, leading ultimately to a reduced endogenous Fe content, and suggesting that overexpression of *COPT* may alter the local response to Fe deficiency in roots.

### Expression of Regulators of Fe Homeostasis in the *COPT^OE^* Seedlings

Certain Fe deficiency responses are under the control of the Cu-responsive SPL7 transcription factor (Kastoori Ramamurthy et al., 2018). To determine whether the inhibition of *IRT1* expression observed in *COPT^OE^* could be a SPL7-mediated response, we checked the expression in *COPT^OE^* seedlings of *SPL7* and *SPL7*-regulated markers of Cu deficiency responses, such as *COPT2* and *FSD1* (**Supplementary Figure S3**), the latter encoding FeSOD (Yamasaki et al., 2009). *SPL7* expression remained mostly unaffected in *COPT^OE^*. Whereas *COPT2* expression was down-regulated in *COPT^OE^* seedlings, *FSD1* expression was not affected under Cu deficiency (**Supplementary Figures S3B, C**). Furthermore, other SPL7 targets were not differentially expressed in the *COPT1^OE^* seedlings under Cu deficiency (**Tables SI** and **SII**), indicating that the SPL7 factor was properly functioning in *COPT^OE^* seedlings and it was not differentially affecting Cu deficiency responses in these plants. On the other hand, the expression of *CSD2*, encoding the chloroplastic Cu/Zn SOD *CSD2*, was reduced in *COPT^OE^* seedlings under Cu sufficiency and excess (**Supplementary Figure S3D**).

**Figure 3:**
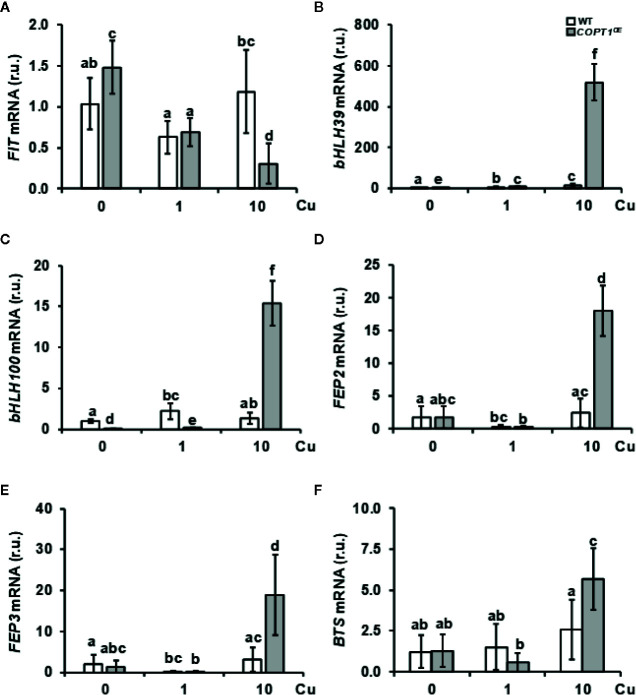
Gene expression of Fe homeostasis regulators in *COPT1^OE^* seedlings. The 7-day-old WT (white bars) and *COPT1^OE^* plants (grey bars) seedlings grown under Cu deficiency (0 μM CuSO_4_), sufficiency (1 μM CuSO_4_) or Cu excess (10 μM CuSO_4_). Expression of *FIT* **(A)**, *bHLH39* **(B)**, *bHLH100* **(C)**, *FEP2* **(D)**, *FEP3* **(E)**, and *BTS* **(F)** was determined by RT-qPCR. Gene expression is represented as relative expression levels (r.u.) in relation to the WT under control conditions. *UBQ10* was used as housekeeping gene. Bars correspond to arithmetic means (2^−ΔΔCt^) ± SD of biological replicates (n = 3). Samples with a letter in common are not significantly different (*p*-value <0.05).

To further address the role of Cu in the lack of Fe-deficiency response, we analyzed the expression of Fe-related bHLH transcription factors and other regulators (**Table 1** and **Figure 3**). A slight increase and a lower expression were observed for *FIT* under Cu deficiency and excess, respectively, in *COPT1^OE^* seedlings compared to the WT (**Figure 3A**). Consistent with *FIT* inhibition under Cu excess, the expression of two of its targets, *IRT1* and *COPT2*, was slightly repressed in *COPT^OE^* seedlings (**Figure 1A** and **Supplementary Figure S3B**), which suggests that high Cu in these plants represses the activity of this master Fe uptake regulator. The expression of the subgroup Ib of Fe-related bHLH (*bHLH38*, *bHLH39*, *bHLH100* and *bHLH101*) transcription factors was also analyzed (**Figures 3B, C**, **Supplementary Figures S4A, B**). We observed that, in the WT plants, expression of *bHLH*-*Ib* factors was in general greater as Cu increased in the growth medium. Remarkably, its expression was highly increased in *COPT1^OE^* seedlings under Cu excess conditions (**Figure 3** and **Supplementary Figure S4**). Therefore, the opposite regulation in *COPT1^OE^* seedlings under Cu excess was observed for *FIT* and the rest of regulatory *bHLH*-*Ib* factors. Whereas *FIT* expression was reduced under Cu excess in *COPT^OE^* respect to the WT, *bHLH*-*Ib* expression was increased under Cu excess (**Figure 3** and **Supplementary Figure S4**).

Among the most induced Fe-related genes in the *COPT1^OE^* line under Cu excess were the Fe peptide regulators *FEP2* and *FEP3* (**Table 1**). The expression of these responds to Fe deficiency in a FIT-independent manner (Hirayama et al., 2018). In agreement with low Fe levels in the *COPT1^OE^* plants, *FEP2* and *FEP3* were clearly induced under Cu excess (**Figures 3D, E**). This result indicated that the FIT-independent signaling pathway for *FEP2* and *FEP3* induction was not affected by deregulated Cu uptake in *COPT1^OE^* under Cu excess.

Taken together these results indicate that strategy I local responses to Fe deficiency cannot take place in *COPT^OE^* seedlings under Cu excess despite the increased expression in *bHLH-Ib* transcription factors and *FEP* genes. This is probably due to the inhibition of *FIT* expression (**Figure 3A**), whereas the opposite occurs under Cu deficiency where, despite slightly enhanced *FIT* expression in the *COPT1^OE^* seedlings, the low *bHLH*-*Ib* expression (**Figure 3** and **Supplementary Figure S4**) was precluding assimilatory Fe deficiency responses.

Regarding to upstream regulators of Fe homeostasis that were repressed in *COPT1^OE^* (**Table 1**), we analyzed the expression of the subgroup IVc members of *bHLH* (*bHLH105/IRL3* and *bHLH115*) transcription factors. *IRL3* and *bHLH115* were repressed in *COPT3^OE^* (**Supplementary Figures S4C, D**). On the other hand, BTS expression encoding an E3 ubiquitin ligase that participates in Fe sensing (Long et al., 2010; Kobayashi et al., 2013) was enhanced under Cu excess in WT plants and further increased in *COPT1^OE^* (**Figure 3F**). This increased expression in BTS and the reduced Fe uptake response under Cu excess (**Figure 1**) was according to the BTS role as a negative regulator of Fe assimilatory responses (Hindt et al., 2017).

Plant hormones are involved in regulating the expression of Fe deficiency-responsive genes (Kobayashi, 2019). In order to check if hormone contents were significantly affected in *COPT1^OE^* seedlings, abscisic acid (ABA), jasmonic acid (JA), and indol acetic acid (IAA) contents were determined (**Supplementary Figure S5**). Whereas the ABA levels were not significantly modified with respect to controls, JA and IAA levels decreased in *COPT1^OE^* seedlings as Cu increased in the medium (**Supplementary Figure S5**).

### Phenotype of *COPT^OE^* Seedlings Under Fe Deficiency

**Figure 4:**
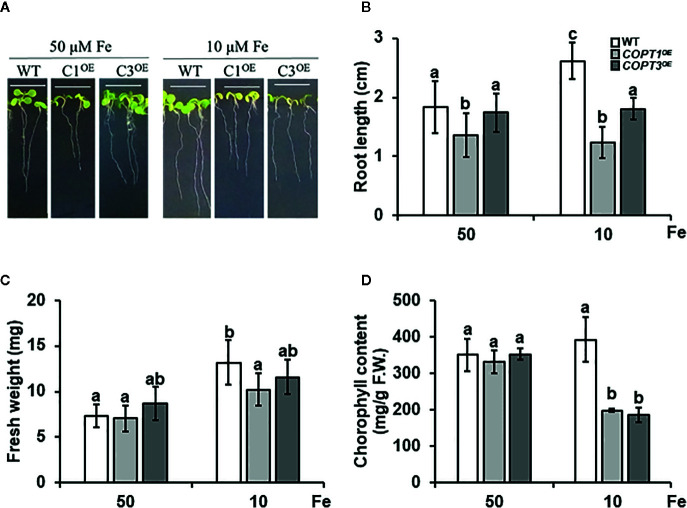
Physiological characterization of *COPT^OE^* seedlings under Fe deficiency. **(A)** Representative photographs of 7-day-old seedlings of WT, *COPT1^OE^* and *COPT3^OE^* under Fe sufficiency (50 μM FeSO_4_) or slight Fe deficiency (10 μM FeSO_4_). White scale bars represent 1 cm. **(B)** Root length of the of WT (white bars), *COPT1^OE^* (light grey bars), and *COPT3^OE^* (dark grey bars) under the same growth conditions as in **(A)**. **(C)** Fresh weight of 5 WT and *COPT1^OE^* seedlings grown under the same conditions as in **(A)**. **(D)** Total chlorophyll content of WT and *COPT^OE^* seedlings grown under the same conditions as in **(A)**. Bars correspond to arithmetic means (2^−ΔΔCt^) ± SD of biological replicates (n = 3). Samples with a letter in common are not significantly different (*p*-value <0.05).

Given the previous data, we studied the phenotype of *COPT^OE^* seedlings under Fe deficiency (**Figure 4**). Thus, 7-day-old WT, *COPT1^OE^*, and *COPT3^OE^* seedlings were grown in media with Fe sufficiency (50 μM FeSO_4_) and slight Fe deficiency (10 μM FeSO_4_) (**Figure 4**). Whereas WT seedlings increased their root length under slight Fe deficiency conditions, *COPT^OE^* presented a similar length of their roots in both media (**Figures 4A, B**). Root shortening in the *COPT^OE^* with respect to WT under slight Fe deficiency was around 50%. No changes were observed in the seedlings fresh weight under sufficiency, while under slight Fe deficiency, the weight was reduced in *COPT1^OE^* (**Figure 4C**). Moreover, the total chlorophyll content did not show significant changes in the WT, while *COPT^OE^* seedlings displayed around 50% lower chlorophyll content in Fe deficiency (**Figure 4D**). Taken together, these data indicate that *COPT^OE^* seedlings are more sensitive to slight Fe deficiency conditions than WT.

Then, we analyzed the expression of Fe homeostasis-related genes under Fe sufficiency and deficiency. *IRT1* expression increased under Fe deficiency in both WT and *COPT^OE^* (**Supplementary Figure S6A**). On the other hand, an increase in *FIT* expression was observed under Fe deficiency in the WT plants, while in *COPT^OE^* this increase was exacerbated (**Supplementary Figure S6B**). Furthermore, *FEP2* and *FEP3* expression was induced in WT plants under Fe deficiency but it was also highly increased in *COPT1^OE^* (**Supplementary Figures S6C, D**). Taken together, these results indicated that *COPT^OE^* seedlings properly responded to Fe deficiency under Cu sufficiency and that their defects in Fe homeostasis were restricted to Cu deficiency and excess conditions.

The expression of the Fe homeostasis regulators *FIT*, *bHLH38*, *bHLH100*, and *UPB1* was analyzed under different metal deficiency and excess conditions in the WT plants (**Supplementary Figure S7**). *FIT*, *bHLH38*, and *bHLH100* where induced under Fe deficiency whereas *UPB1* was repressed. Curiously, all of them but *FIT* were regulated in the same sense under Cu excess and Fe deficiency, either induced (*bHLH38* and *bHLH100*) or repressed (*UPB1*), suggesting that maybe there are certain similarities or common steps in signal transduction between the responses to Cu excess and Fe deficiency, leading to the induction of these transcriptional factors.

### Expression of Genes Involved in Tetrapyrrole Biosynthesis in *COPT^OE^* Seedlings

**Figure 5:**
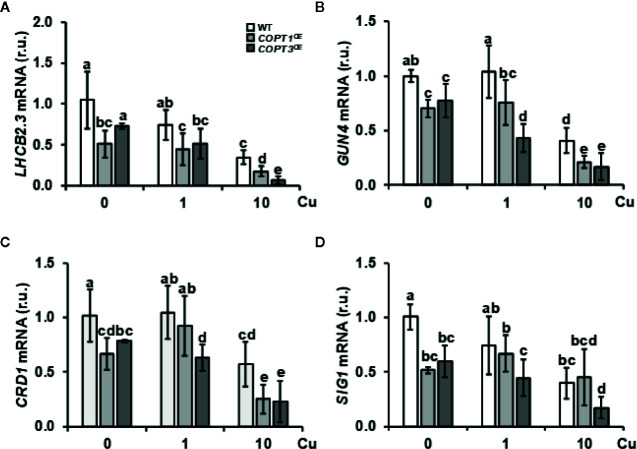
Expression of genes related to tetrapyrrole biosynthesis and retrograde signaling in *COPT^OE^*. Expression of the *LHCB2.3* **(A)**, *GUN4* **(B)**, *CDR1* **(C)**, and *SIG1A* **(D)** genes in WT (white bars), *COPT1^OE^* (light grey bars), and *COPT3^OE^* (dark grey bars) seedlings grown under Cu deficiency (0 μM CuSO_4_), sufficiency (1 μM CuSO_4_) or Cu excess (10 μM CuSO_4_) was determined by RT-qPCR. Gene expression is represented as relative expression levels (r.u.) in relation to the WT under control conditions. *UBQ10* was used as housekeeping gene. Bars correspond to arithmetic means (2^−ΔΔCt^) ± SD of biological replicates (n = 3). Samples with a letter in common are not significantly different (*p*-value <0.05).

GO analysis of the global transcriptomic analysis revealed that the chloroplast was the subcellular compartment most affected in *COPT1^OE^* (**Supplementary Table SV**). GO categories differentially enriched among genes differentially regulated in *COPT1^OE^* seedlings were photosynthesis-associated nuclear genes (*PhANG*) and genes related to photomorphogenesis, and oxidative stress (**Table 1** and **Supplementary Tables SIII** and **SV**). Therefore, we decided to assess the alteration of the expression of genes in the tetrapyrrole biosynthesis pathway and the retrograde signaling components (**Table 1**). The *LHCB2.3* expression, a representant among the multiple *PhANG*, which are down-regulated in the *COPT1^OE^* seedlings (**Table SII**), was confirmed to be repressed (**Figure 5A**). Moreover, the expression of *GUN4*, a regulator of the plastidic tetrapyrrole signaling (Davison et al., 2005), was also repressed in most conditions (**Figure 5B**). In addition, *CHL27/CDR1* (Bang et al., 2008) was also down-regulated in *COPT^OE^* seedlings (**Figure 5C**), further confirming the involvement of tetrapyrrole signaling in Cu-dependent Fe deficiency responses. The regulation of a chloroplastic sigma factor *SIG1* involved in retrograde signaling (Macadlo et al., 2019) was also down-regulated under Cu deficiency in *COPT^OE^* seedlings (**Figure 5D**). Taken together, these results indicated that the tetrapyrrole signaling pathway was affected under both Cu deficiency and excess, probably as a consequence of the common Fe deficiency conditions faced by *COPT^OE^* seedlings.

### Root Respiration and Oxidative Stress in *COPT^OE^* Seedlings

**Figure 6:**
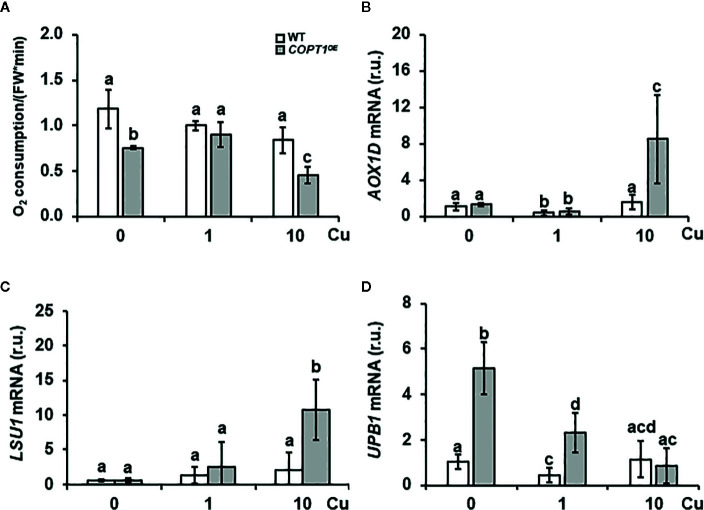
Root O_2_ consumption and expression of stress-related genes in *COPT1^OE^*. **(A)** O_2_ consumption in roots of 10-day-old WT and *COPT1^OE^* seedlings cultivated with different CuSO_4_ concentrations. The rate of O_2_ decrease was referenced to roots F.W. (nmol O_2_/OD600 * F.W). The values represented are the arithmetic mean ± SD of n = 3 biological replicates. Expression of the *AOX-1D* **(B)**, *LSU1* **(C)**, and *UPB1* **(D)** genes in WT (white bars) and *COPT1^OE^* (grey bars) seedlings grown under Cu deficiency (0 μM CuSO_4_), sufficiency (1 μM CuSO_4_) or Cu excess (10 μM CuSO_4_) was determined by RT-qPCR. Gene expression is represented as relative expression levels (r.u.) in relation to the WT under control conditions. *UBQ10* was used as housekeeping gene. Bars correspond to arithmetic means (2^−ΔΔCt^) ± SD of biological replicates (n = 3). Samples with a letter in common are not significantly different (*p*-value <0.05).

Since the respiratory electron transport chain is one of the most metal requiring processes, O_2_ consumption was measured in 7-day-old roots of WT and *COPT1^OE^* seedlings grown in deficiency (0 μM CuSO_4_), sufficiency (1 μM CuSO_4_) and excess CuSO_4_ (10 μM CuSO_4_) (**Figure 6A**). The results showed that O_2_ consumption in WT seedlings did not exhibit significant changes between the three media, while *COPT1^OE^* displayed lower O_2_ consumption when the Cu conditions were not optimal, in both deficiency and in excess, with a decrease of 42 and 46% respectively, compared to WT (**Figure 6A**). In agreement with a defective functioning of the respiratory electron transport chain, expression of the *ALTERNATIVE OXIDASE 1D* (*AOX-1D*), aimed to restore electron flow in a non-phosphorylating bypass (Clifton et al., 2006; Selinski et al., 2018), was increased in *COPT1^OE^* (**Figure 6B**). Moreover, other *AOX* genes, such as *AOX1A* (+2.46) and *AOX2* (+2.08), were also induced under Cu excess in *COPT^OE^* (**Supplementary Table SI**). On the other hand, expression of the *LOW SULPHUR UPREGULATED1* (*LSU1*) has been shown to prevent chloroplastic ROS production by interacting with *FSD2* (Garcia-Molina et al., 2017). *LSU1* was also greatly induced under Cu excess (**Figure 6C**), as well as *LSU2* and *LSU3* (**Supplementary Table SI**). Inhibition of *FSD1* and *CSD2* expression in the *COPT^OE^* plants (**Supplementary Figure S3**) suggested a defective antioxidant capacity by an increase in the radical superoxide (O_2_ **^.−^**) and a decrease in hydrogen peroxide (H_2_O_2_).

UPB1 spatially regulates ROS distribution in the root transition zone where the balance between O_2_ **^.−^** and H_2_O_2_ controls the transition between root cell proliferation and differentiation (Tsukagoshi et al., 2010). *UPB1* expression was induced under Cu deficiency and sufficiency in *COPT1^OE^* seedlings compared to WT (**Figure 6D**). Accordingly, two of its direct targets, *PER39* and *PER40*, were also up-regulated under Cu deficiency (**Supplementary Figures S8A, B**). Furthermore, *UBP1* expression under different metal stress conditions in the WT indicated that was repressed under Cu excess as well as when Fe is low (**Supplementary Figure S7D**). These responses indicated clearly different responses of Fe regulators to low and high Cu levels.

Since these results would lead to a further decrease in H_2_O_2_, seedlings were grown in Cu-deficient media in the presence of H_2_O_2_ and the reductants ascorbic acid (AsH) and 1,4-ditiothreitol (DTT) (**Supplementary Figure S9**). *COPT1^OE^* seedlings showed shorter roots than controls under Cu deficiency. Whereas no clear effects were observed on root length for the H_2_O_2_ treatment, reductants such as AsH and DTT, improved the root growth of *COPT1^OE^* compared to WT (**Supplementary Figure S9**) further pointing to increased oxidative stress in the overexpressing line under Cu deficiency.

**Figure 7:**
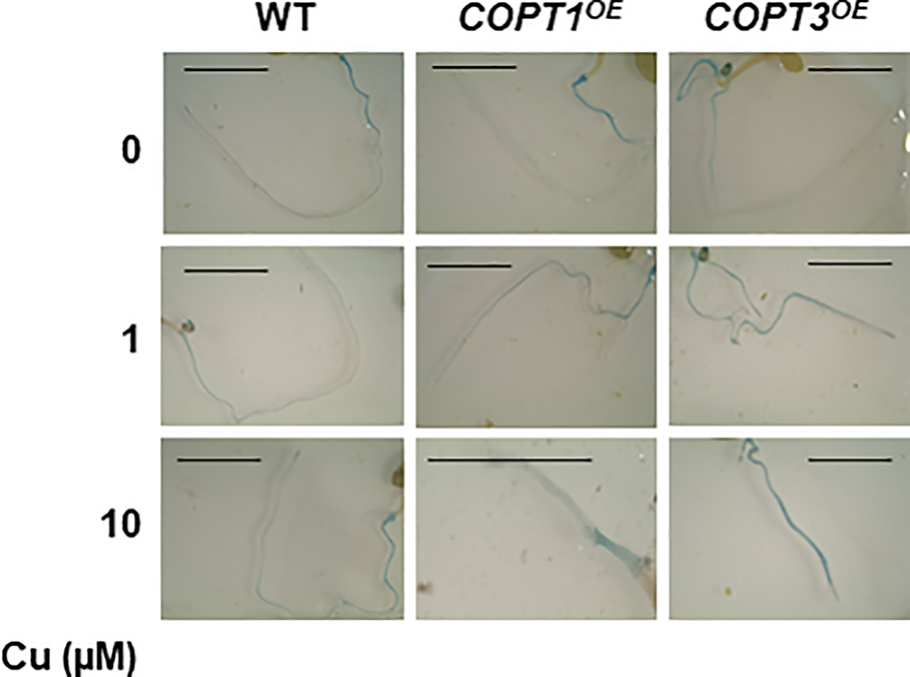
Detection of Fe^3+^ in *COPT^OE^* roots. Seven-day old roots of WT, *COPT1^OE^* and *COPT3^OE^* seedlings grown under Cu deficiency (0 μM CuSO_4_), sufficiency (1 μM CuSO_4_) or Cu excess (10 μM CuSO_4_) were stained with Perl´s blue to detect Fe^3+^. Scale bars represent 1 cm.

Additionally, since the Fe content was lower in *COPT^OE^*, a Fe excess treatment was also provided with no differential effect compared to controls (**Supplementary Figure S9**). In order to ascertain the Fe redox state in the roots, 7-day-old seedlings grown in the same media with different Cu content (0, 1, and 10 μM CuSO_4_) were stained with the Perl´s blue to detect the presence of Fe^3+^. The intensity of stained roots indicated that Fe^3+^ increased as Cu rise in the media from 0 to 10 μM Cu in all seedlings´ genotypes. Moreover, *COPT^OE^* seedlings were more intensely stained than WT in all the conditions (**Figure 7**) and this result is reproducible in 11-day-old seedlings (**Supplementary Figure S10**), indicating that Cu^+^ uptake through COPT is affecting the redox state of incorporated Fe leading to an increase in the Fe^3+^/Fe^2+^ ratio in the *COPT^OE^* roots since the lower total Fe concentration (**Figure 1D**).

**Figure 8:**
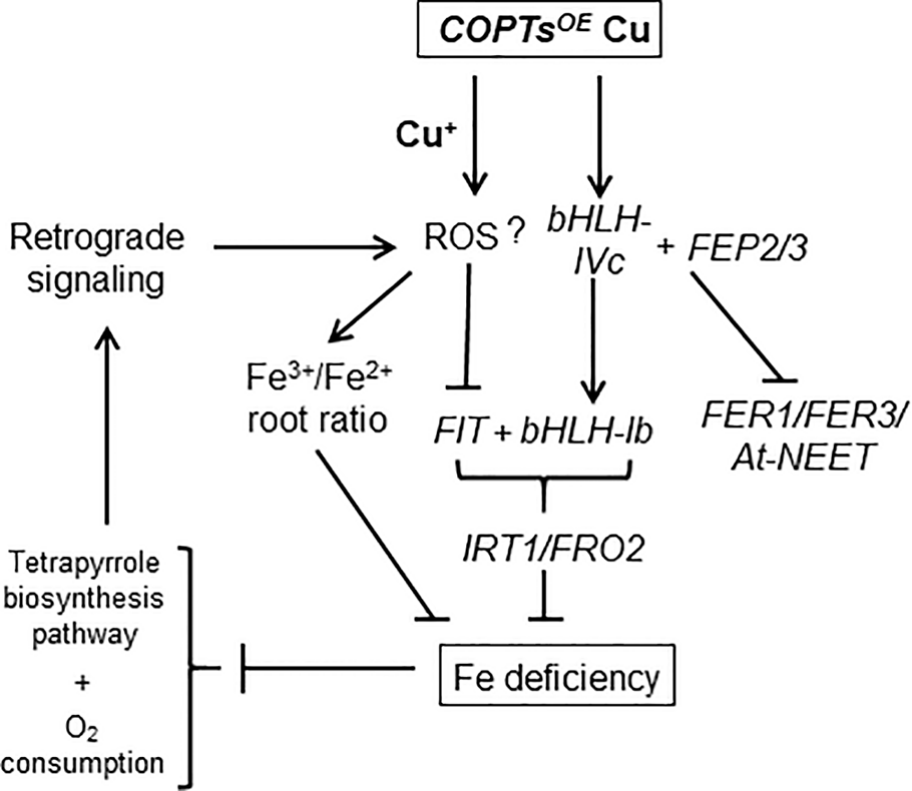
Genetic model of the Cu effects on Fe homeostasis in *COPT^OE^*. Deregulated Cu^+^ entrance by constitutive COPT overexpression under high Cu increased the Fe^3+^/Fe^2+^ ratio in roots. *COPT^OE^* displayed Fe deficiency symptoms probably as a consequence of the lack of Fe assimilatory response (*IRT1/FRO2*), since under high Cu conditions *COPT^OE^* plants activated the expression of the *bHLH-Ib* factors but not *FIT*. Cu excess increased ROS that could activate *FEP2/FEP3* expression, which up-regulate *bHLH-Ib* factors. *BTS* affected bHLH-IVc stability and expression of its target genes (*FER1*, *FER3* and *At-NEET*). Fe deficiency affected tetrapyrrole biosynthesis pathway and O_2_ consumption that could further increase ROS production and inhibit Fe uptake through retrograde signaling.

These results suggested that the reduction in Fe content observed in *COPT^OE^* could be due to the increased Fe^3+^/Fe^2+^ ratio when Cu^+^ entrance was enhanced and deregulated in *COPT^OE^* seedlings. Thus, a model for the Cu excess effects on Fe homeostasis in *COPT^OE^* seedlings has been proposed (**Figure 8**). The deregulated Cu^+^ entrance in *COPT^OE^* plants under high Cu activated the expression of the *bHLH-Ib* factors but not *FIT*. This effect redounded in Fe deficiency symptoms in *COPT^OE^* probably as a consequence of the lack of Fe assimilatory response. Cu excess increased the Fe^3+^/Fe^2+^ ratio in *COPT^OE^* roots, probably leading to signaling pathways that could further inhibit Fe uptake.

## Discussion

In this report, we studied the effects of deregulated Cu^+^ entrance in *COPT^OE^* seedlings, which overexpress COPT Cu transporters (Andrés-Colás et al., 2010), on global gene expression when seedlings were grown in Cu concentrations within the physiological range that plants encounter in their natural environment. Our results indicate that Fe transport and homeostasis were altered in *COPT^OE^*. Despite containing low Fe, *COPT^OE^* was not able to orchestrate an optimal local Fe-uptake response at their roots under both Cu deficiency and excess (**Figure 1**). The causes for the Cu interference with the Fe-uptake response seem different in both conditions. Under Cu deficiency, *COPT^OE^* induced *FIT*, but the expression of subgroup Ib of *bHLH* remained low. On the contrary, under Cu excess, *FIT* was repressed, despite high *FEP*s and *bHLH*-*Ib* expression (**Figure 3**). Based on the necessity of expressing both FIT and subgroup Ib of bHLH transcription factors for Fe-uptake responses (Yuan et al., 2008; Wang et al., 2013), *COPT^OE^* seedlings were unable to properly respond to Fe deficiency under non-optimal Cu supply.

Fe and Cu homeostasis interact at different levels in plants (Puig et al., 2007; Bernal et al., 2012; Perea-García et al., 2013; Kastoori Ramamurthy et al., 2018). For instance, high Cu concentrations in the media lower leaf Fe content (Waters et al., 2012; Waters and Armbrust, 2013). In our experimental conditions, a slight decrease in *IRT1* expression was observed in the WT seedlings under Cu excess, which was further exacerbated in *COPT^OE^*. On the other hand, higher Cu content was observed under Fe deficiency in WT (Kastoori Ramamurthy et al., 2018). Among the reasons for increased Cu content under Fe deficiency are the increased uptake of both the unspecific Cu^2+^ entrance through ZIP transporters and the specific Cu^+^ entrance through *COPT2* (Colangelo and Guerinot, 2004; Perea-García et al., 2013). Nevertheless, the reason for Cu^+^ requirement under Fe deficiency remains unsolved.

Although recent data suggested that Fe deficiency responses are under the control of SPL7 (Kastoori Ramamurthy et al., 2018), Cu-dependent SPL7-mediated responses did not seem to be the cause of *IRT1* down-regulation in *COPT^OE^* since the expression of other SPL7 target genes was not significantly affected in these plants. On the other hand, different hormones are involved in modulating Fe deficiency responses in plants (Kobayashi, 2019). JA has been shown to inhibit the expression of *bHLH-Ib* and promote FIT degradation, resulting in reduced expression of the Fe uptake genes *IRT1* and *FRO2* and increased sensitivity to Fe deficiency (Cui et al., 2018). Since JA is a negative regulator of Fe deficiency responses and JA content was reduced in *COPT1^OE^*, lower levels of JA could not justify the decrease in Fe uptake under our experimental conditions. However, auxins can enhance *FIT* and *FRO2* expression (Chen et al., 2010), and, in this sense, we cannot discard that IAA decreased levels in *COPT1^OE^* seedlings under Cu excess could at least partially account for the low *FIT* expression in *COPT1^OE^* plants.

The oxidative stress produced by metal deficiencies and excess could also account for the Fe and Cu crosstalk (Ravet and Pilon, 2013). Indeed, Cu phytotoxicity has been attributed to increased H_2_O_2_ production (Cuypers et al., 2010) and the deficiency of Cu affects the functioning of the respiratory and photosynthetic electron transport chains, which increases the production of the O_2_ **^.−^** (Abdel-Ghany et al., 2005; Yamasaki et al., 2009). Therefore, the majority type of ROS could be different under conditions of metal deficiency or excess. Increased oxidative stress leading to lipid peroxidation has been previously reported to occur in *COPT^OE^* seedlings (Andrés-Colás et al., 2010). According to the slightly increased Cu content in *COPT^OE^* seedlings (Andrés-Colás et al., 2010), they should have increased *CSD2* levels under Cu excess, instead of the observed lower levels. This result suggests a difficulty of the *COPT^OE^* chloroplasts in detoxifying O_2_ **^.−^** under Cu excess, since they cannot completely activate neither *FSD1* nor *CSD2* compared to the WT, maybe redounding in increased oxidative stress. The induction of several members of the LSU family (*LSU1*, *LSU2* and *LSU3*) under Cu excess in the *COPT^OE^* plants is in agreement with their induction under sulfur deficiency and by Fe deficiency and Cu excess (Garcia-Molina et al., 2017). LSU1 interacts with chloroplastic FSD2 and stimulates its enzymatic activity *in vivo* and *in vitro* conforming a hub in coordinating plant responses to a wide spectrum of abiotic stress conditions (Garcia-Molina et al., 2017). Moreover, *COPT1^OE^* seedlings showed higher Cu-induced K^+^ efflux and net Ca^2+^ influx at the root tip level compared to the WT (Rodrigo-Moreno et al., 2013), although no significant changes of the membrane potential were detected upon Cu addition (Sanz et al., 2019).

Although increased O_2_ consumption has been also reported in sugar beet and cucumber roots (Lopez-Millan et al., 2000; Vigani et al., 2009), the ability to consume O_2_ decreased in *COPT1^OE^* roots under both Cu deficiency and excess (**Figure 6A**). High Cu in *COPT1^OE^* triggers a response that includes the expression of genes related to mitochondrial stress (Arnholdt-Schmitt et al., 2006; Selinski et al., 2018), such as *AOX1A*, *AOX1D* and *AOX2*. Results shown here support the respiratory shield hypothesis, where the mitochondrial Fe proteins are necessary to maintain high O_2_ consumption in the respiratory electron transport chain contributing to the microaerobic environment necessary to maintain a Fe^2+^ pool. The problem due to lack of respiratory shield could be aggravated in conditions of Fe deficiency and high and temporarily deregulated Cu^+^ entry. In this sense, the defect of Fe/S and heme proteins will lead to defective respiratory shield in strains with high metabolism (López-Torrejón et al., 2016; Wofford et al., 2019), aggravated in *COPT1^OE^* seedlings by deregulated Cu^+^ uptake. In any case, our observations support that optimal Cu supply is required for normal O_2_ consumption in *COPT1^OE^* plants.

Cu deficiency affects Fe homeostasis specifically causing a defect in root-to-shoot translocation, which has been attributed to a decrease in Cu-dependent ferroxidase activity (Bernal et al., 2012; Waters and Armbrust, 2013). Assuming that a certain Fe^3+^/Fe^2+^ and Cu^2+^/Cu^+^ intracellular ratios are necessary for uptake/mobilization processes, Cu^+^ uptake could affect the Fe^3+^/Fe^2+^ ratio at different levels. Firstly, due to its lower standard reduction potential, Cu^+^ could directly reduce Fe^3+^ to Fe^2+^. However, this is not the case since increased Fe^3+^ was observed in *COPT^OE^* seedlings (**Figure 7**). Moreover, highly reactive, cytosolic free Cu^+^ is almost absent in the cytosol (Rae et al., 1999). Instead, increased oxidative stress provoked by deregulated Cu^+^ entrance (Rodrigo-Moreno et al., 2013) could influence intracellular general redox status, leading to Fe oxidation. On the other hand, Cu deficiency would facilitate Fe^2+^ incorporation through the enhanced expression of *FRO* metalloreductases and inhibit Cu-dependent ferroxidase activity (Bernal et al., 2012; Waters et al., 2012; Waters and Armbrust, 2013). Since the Fe^3+^/Fe^2+^ ratio depends on the relative ferroxidase versus ferroreductase activities, an increased Fe^3+^/Fe^2+^ ratio would be expected in the presence of excess Cu. The other way around, the Fe^3+^/Fe^2+^ ratio should be decreased under Cu deficiency. Under our experimental conditions, we were not able to detect a significant change in ferroreductase activity and just a slight decrease is observed in *COPT^OE^* seedlings under Cu excess (**Figure 1B**), questioning this activity being the only cause of the Fe deficiency. Finally, Cu uptake increased Fe^3+^ in the roots, probably by means of the oxidative conditions created. As a consequence, decreased Fe mobilization in roots could lead to Fe deficiency in leaves. In this sense, Cu excess and deregulated Cu^+^ uptake in *COPT^OE^* seedlings increased the Fe^3+^/Fe^2+^ ratio leading to Fe deficiency effects, being it at least one of the problems faced by Cu^+^ toxicity (**Figure 8**). Accordingly, this suggestion could explain previous results where plants grown without Fe were more susceptible to Cu toxicity (Waters and Armbrust, 2013).

In order to address how Cu influenced the signaling pathways involved in the Fe deficiency response, the expression of *BTS* and several other genes encoding RING E3 ubiquitin ligases were induced in the *COPT1^OE^* seedlings. This result points to the putative Fe-sensing BTS (Selote et al., 2015), and maybe other RING E3 ubiquitin ligases, as candidates in upstream sensing of the lack of Fe mobilization in *COPT1^OE^* plants under mild Cu excess. On the other hand, our results indicated that tetrapyrrole signaling was affected under both Cu deficiency and excess, probably as a consequence of the common Fe deficiency conditions faced by *COPT^OE^* seedlings (**Supplementary Tables SIII** and **SIV**). Most of the genes involved in tetrapyrrole metabolism showed synchronized and light-dependent expression patterns (Matsumoto et al., 2004). Multiple *PhANG* were down-regulated in *COPT1^OE^* under both Cu deficiency and excess in addition to several regulators. Curiously, *CHL27/CRD1* (*COPPER RESPONSE DEFECT1*) was first characterized in a screening for Cu conditional phenotypes in *Chlamydomonas* where Cu scarcity in plastocyanin is counteracted by a heme-containing cytochrome (Moseley et al., 2000), although this substitution has not been shown to occur in *Arabidopsis*. Moreover, the GUN4 porphyrin-binding protein enhances Mg-chelatase activity (Davison et al., 2005) and its repression under Cu excess could play a role in the control of substrate flow into the heme or chlorophyll branch (Tanaka and Tanaka, 2007). On the other hand, nuclear-encoded sigma factor, such as SIG1, could be involved in the integration of light and circadian signals that regulate chloroplast transcription (Belbin et al., 2017). These results underscore the importance of tightly regulated Cu homeostasis at the spatial–temporal level, in order to orchestrate an optimal Fe distribution and to avoid oxidative damage to highly sensitive Fe-dependent processes, such as Fe/S cluster assembly and tetrapyrrole biosynthesis. It is tempting to speculate that Fe and other metals, which affect at the cellular redox state and either participate or interfere with sensitive processes, have to be subjected to differential diurnal variation in the expression patterns of their homeostatic components (Peñarrubia et al., 2015; Zhang and Krämer, 2018). In this sense, the induced expression of *UPB1* in *COPT^OE^* plants under Cu deficiency could emphasize the requirement of spatially appropriate Cu uptake at the root tip, where normally *COPT1* is expressed (Sancenón et al., 2003), to avoid oxidative interference along the root with Fe translocation to the aerial part (Tsukagoshi et al., 2010).

Taken together, these results indicated that high Cu renders increased Fe^3+^ in the root. Despite low Fe in the shoot, the presence of high Fe^3+^/Fe^2+^ ratio in the root prevented local responses to Fe deficiency (**Figure 8**). The understanding of Cu influence on Fe mobilization and redistribution in the plant could help to ameliorate field treatments to maximize crop production under Fe deficiency through optimizing Cu homeostasis.

## Data Availability Statement

The datasets generated for this study can be found in the: The microarray raw data were deposited in the NCBI’s Gene Expression Omnibus and are accessible through GEO Series accession number GSE143857.

## Author Contributions

LP and SP conceived the idea and wrote the manuscript. MP-A and FV-S performed the *COPT1^OE^* global analysis. AP-G and AA-B performed the physiological and molecular experiments in mutant plants. All authors contributed to the article and approved the submitted version.

## Funding

This work was supported by grant BIO2017-87828-C2-1-P from the Spanish Ministry of Economy and Competitiveness, and by FEDER funds from the European Union.

## Conflict of Interest

The authors declare that the research was conducted in the absence of any commercial or financial relationships that could be construed as a potential conflict of interest.
