# Supplementary material for: Deregulated High Affinity Copper Transport Alters Iron Homeostasis in Arabidopsis
Source: Front Plant Sci. 2020 Jul 23;11:1106. doi: 10.3389/fpls.2020.01106 (PMC7390907; doi:10.3389/fpls.2020.01106)
Supplement: Supplementary file 1 [file DataSheet_1.docx]

Supplementary Material

# Supplementary Figures

**Figure S1**. Overview of the *COPT1^OE^* transcriptome. Venn diagrams of genes induced and repressed in *COPT1^OE^* seedlings in deficiency and excess of Cu. WT and *COPT1^OE^* seedlings grown in ½ MS for 7 days (½ MS) and in ½ MS supplemented with 10 μM CuSO_4_ (½ MS + 10 Cu). The values ​​correspond to the number of genes induced or repressed in *COPT1^OE^* seedlings after the microarray analysis in the two Cu conditions tested.


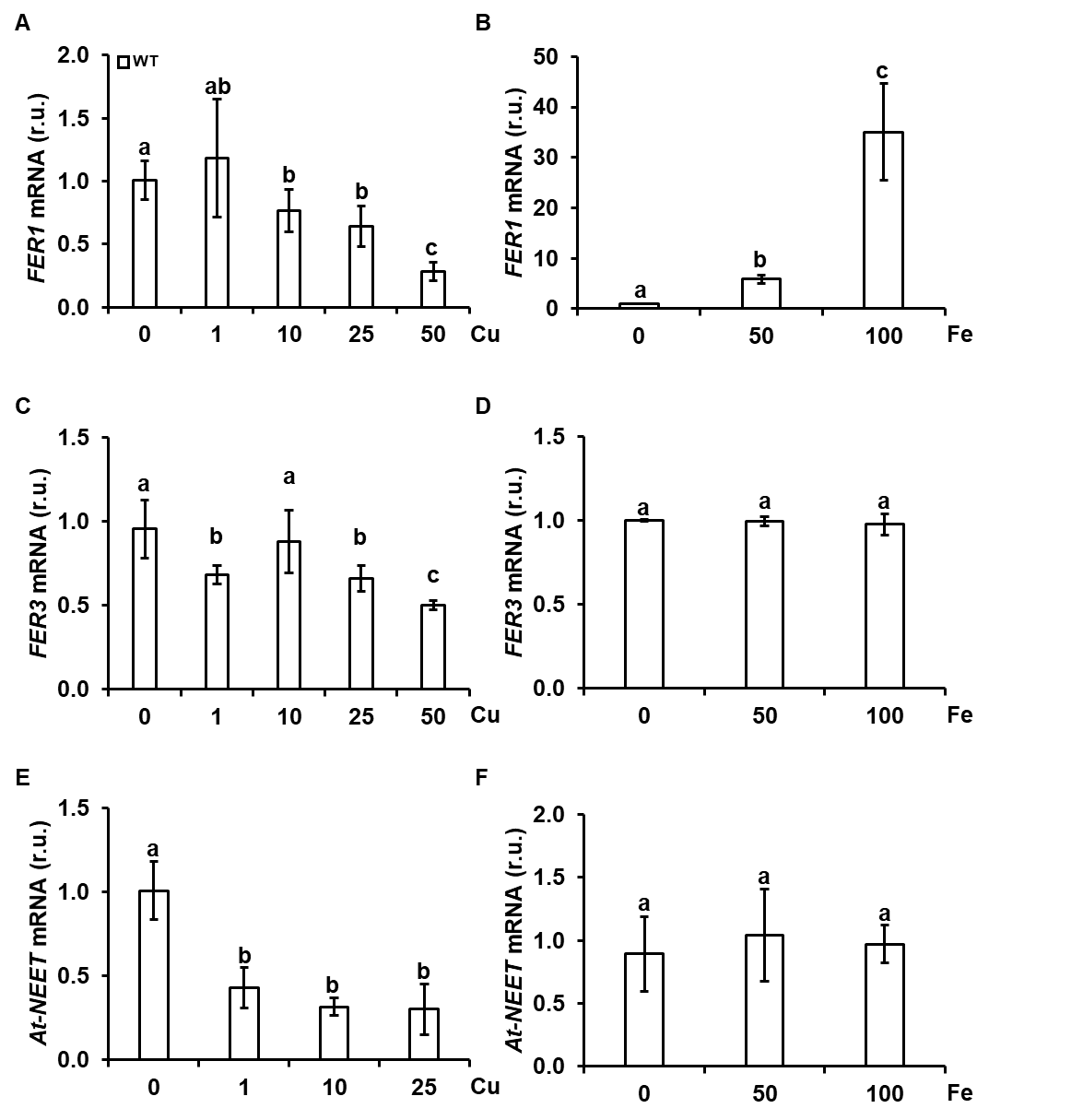


**Figure S2.** Expression of Fe metabolism genes under different metal concentration. Relative expression of *FER1* **(A, B)**, *FER3* **(C, D),** and *At-NEET* **(E, F)** in WT seedlings grown under different Cu content (0, 1, 10, 25, and 50 μM CuSO_4_) **(A, C, E)** or Fe content (0, 50 and 100 μM FeSO_4_) **(B, D, F)**. The mRNA levels are expressed as relative expression levels (r.u.) in relation to the WT under control conditions. *UBQ10* was used as housekeeping gene. Bars correspond to arithmetic means (2^-∆∆Ct^) ± standard deviation (SD) of biological replicates (n=3). For each particular gene, samples with a letter in common are not significantly different (P < 0.05).

**Figure S3.** Expression of Cu-related genes in *COPT^OE^* seedlings. Relative expression levels of the *SPL7* **(A)** *COPT2* **(B)***, FSD1* **(C)** and *CSD2* **(D)** genes in WT and *COPT^OE^* seedlings grown under Cu deficiency (0 μM CuSO_4_), sufficiency (1 μM CuSO_4_) or Cu excess (10 μM CuSO_4_). The mRNA levels are expressed as relative expression levels (r.u.) in relation to the WT under control conditions. *UBQ10* was used as housekeeping gene. Bars correspond to arithmetic means (2^-∆∆Ct^) ± standard deviation (SD) of biological replicates (n=3). For each particular gene, samples with a letter in common are not significantly different (P < 0.05).


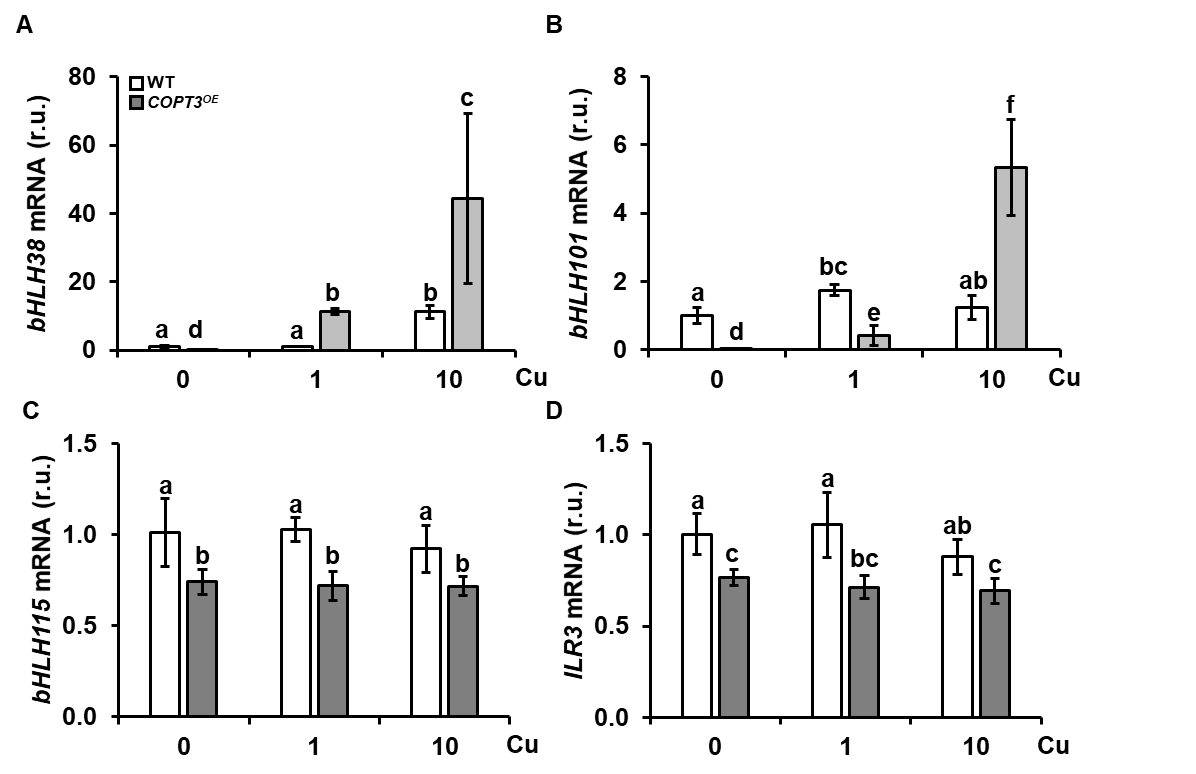


**Figure S4.** Expression of Fe-related *bHLH-Ib* and *bHLH-IVc* in *COPT3^OE^* seedlings. Expression of the *bHLH38* **(A)***, bHLH101* **(B)***, bHLH115* **(C),** and *IRL3* **(D)** genes in 7-day-old WT (white bars) and *COPT3^OE^* (grey bars) seedlings grown under Cu deficiency (0 μM CuSO_4_), sufficiency (1 μM CuSO_4_) or Cu excess (10 μM CuSO_4_). The mRNA levels are expressed as relative expression levels (r.u.) in relation to the WT under control conditions. *UBQ10* was used as housekeeping gene. Bars correspond to arithmetic means (2^-∆∆Ct^) ± standard deviation (SD) of biological replicates (n=3). For each particular gene, samples with a letter in common are not significantly different (P < 0.05).

**Figure S5.** Hormone concentrations in *COPT1^OE^* grown under different Cu content. ABA **(A)**, JA **(B)** and IAA **(C)** concentrations were determined in lyophilized samples and analyzed by UHPLC. Bars correspond to mean ± standard deviation (SD) of biological replicates (n=3). Statistical differences (P < 0.05) between the values of the WT and *COPT1^OE^* in each condition are indicated by an asterisk (*).

**B**

**A**

**Fe**

**Fe**

**D**

**C**


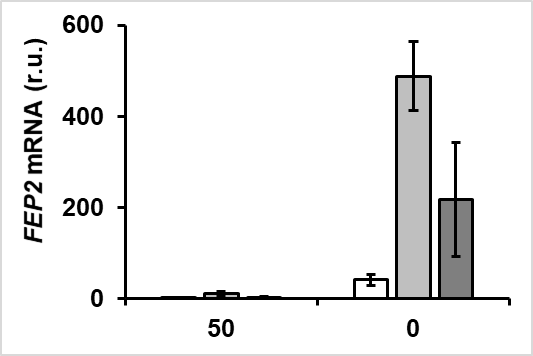


**Fe**

**Fe**


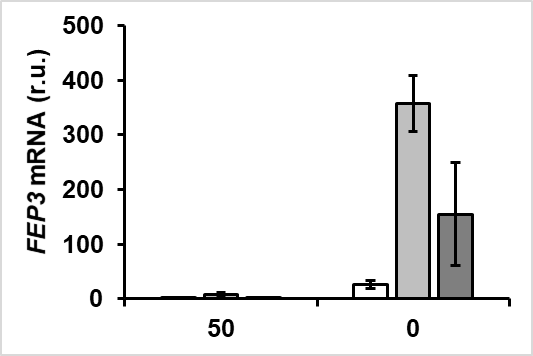


**a**

**b**

**c**

**d**

**e**

**f**

**a**

**b**

**c**

**d**

**e**

**f**


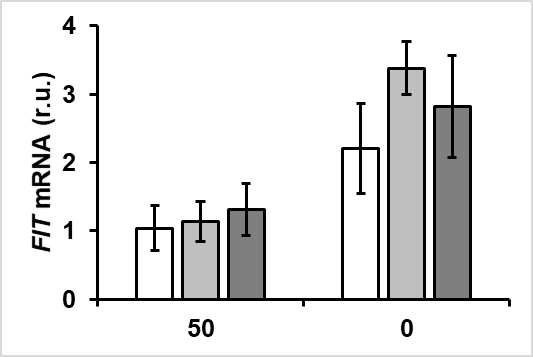


**a**

**a**

**a**

**b**

**b**

**b**


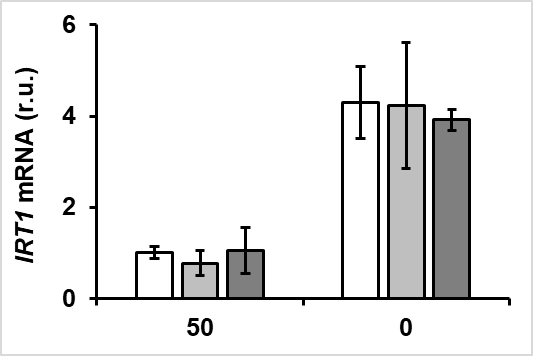


**a**

**b**

**ab**

**c**

**c**

**c**

**WT**

***COPT1^OE^***

***COPT3^OE^***

**Figure S6.** Expression of Fe homeostasis genes in *COPT1^OE^* seedlings. *IRT1* **(A)**, *FIT* **(B)**, *FEP2* **(C)** and *FEP3* **(D)** expression in 7-day-old WT (white bars) and *AtCOPT1^OE^* (grey bars) seedlings grown under Fe deficiency (0 μM FeSO_4_) or Fe sufficiency (50 μM FeSO_4_) was determined by RT-qPCR. *UBQ10* was used as housekeeping gene. Bars correspond to arithmetic means (2^-∆∆Ct^) ± standard deviation (SD) of biological replicates (n=3). Samples with a letter in common are not significantly different (P < 0.05).


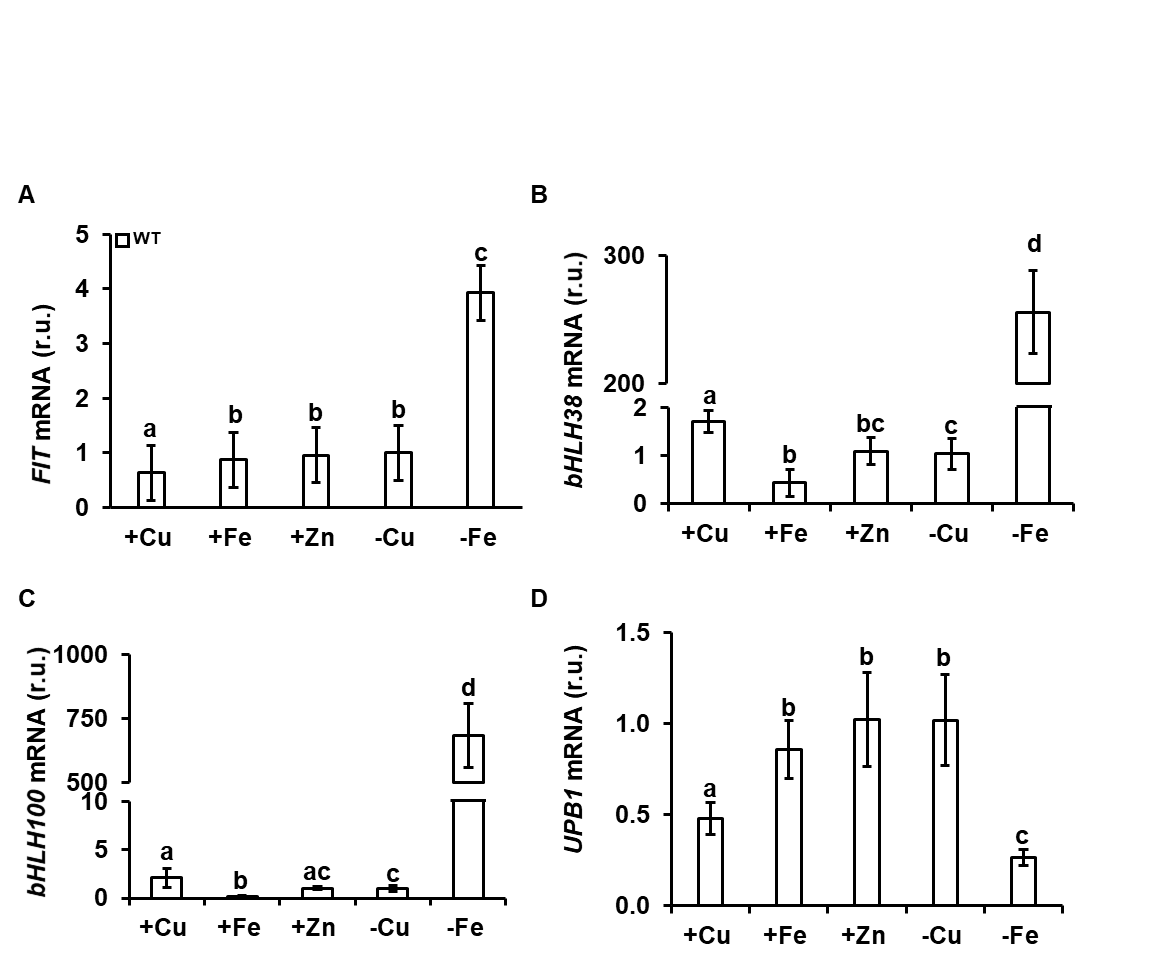


**Figure S7.** Expression of Fe regulatory genes on different metal deficiency and excess. Expression of *FIT* **(A)**, *bHLH38* **(B)***, bHLH100* **(C),** and *UPB1* **(D)** in WT (white bars) seedlings grown under Cu, Zn and Fe excess (sufficiency + 30 μM) or Cu and Fe deficiency (0 μM) was determined by RT-qPCR. The mRNA levels are expressed as relative expression levels (r.u.) in relation to the WT under control conditions. *UBQ10* was used as housekeeping gene. Bars correspond to arithmetic means (2^-∆∆Ct^) ± standard deviation (SD) of biological replicates (n=3). Samples with a letter in common are not significantly different (P < 0.05).

**Figure S8.** Expression of peroxidase genes in *COPT1^OE^* seedlings. Expression of *PER39* **(A)** and *PER40* **(B)** in WT (white bars) and *COPT1^OE^* (grey bars) seedlings grown under Cu deficiency (0 μM CuSO_4_) or sufficiency (1 μM CuSO_4_) was determined by RT-qPCR. The mRNA levels are expressed as relative expression levels (r.u.) in relation to the WT under control conditions. *UBQ10* was used as housekeeping gene. Bars correspond to arithmetic means (2^-∆∆Ct^) ± standard deviation (SD) of biological replicates (n=3). Samples with a letter in common are not significantly different (P < 0.05).


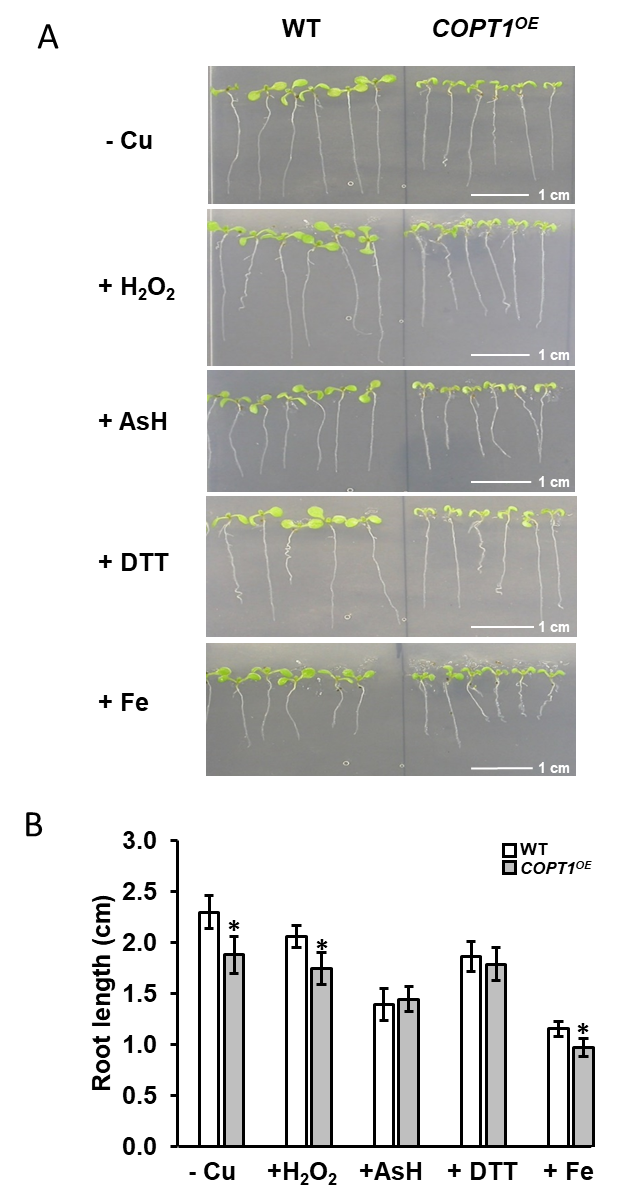


**Figure S9.** Phenotype of *COPT1^OE^* seedlings under Cu deficiency with different compounds. **(A)** Representative photographs of 7-day-old seedlings of WT and *COPT1^OE^* under Cu deficiency (0 μM CuSO_4_) or H_2_O_2_ (100 μM), AsH (100 μM), DTT (100 μM) or Fe excess (500 μM FeSO_4_). **(B)** Root length of the of WT and *COPT1^OE^* seedlings under the same growth conditions as in (A). The values represent the mean ± standard deviation (SD) of biological replicates (n=3). Statistical differences (P < 0.05) are indicated by an asterisk (*). Scale-bars represent 1 cm.


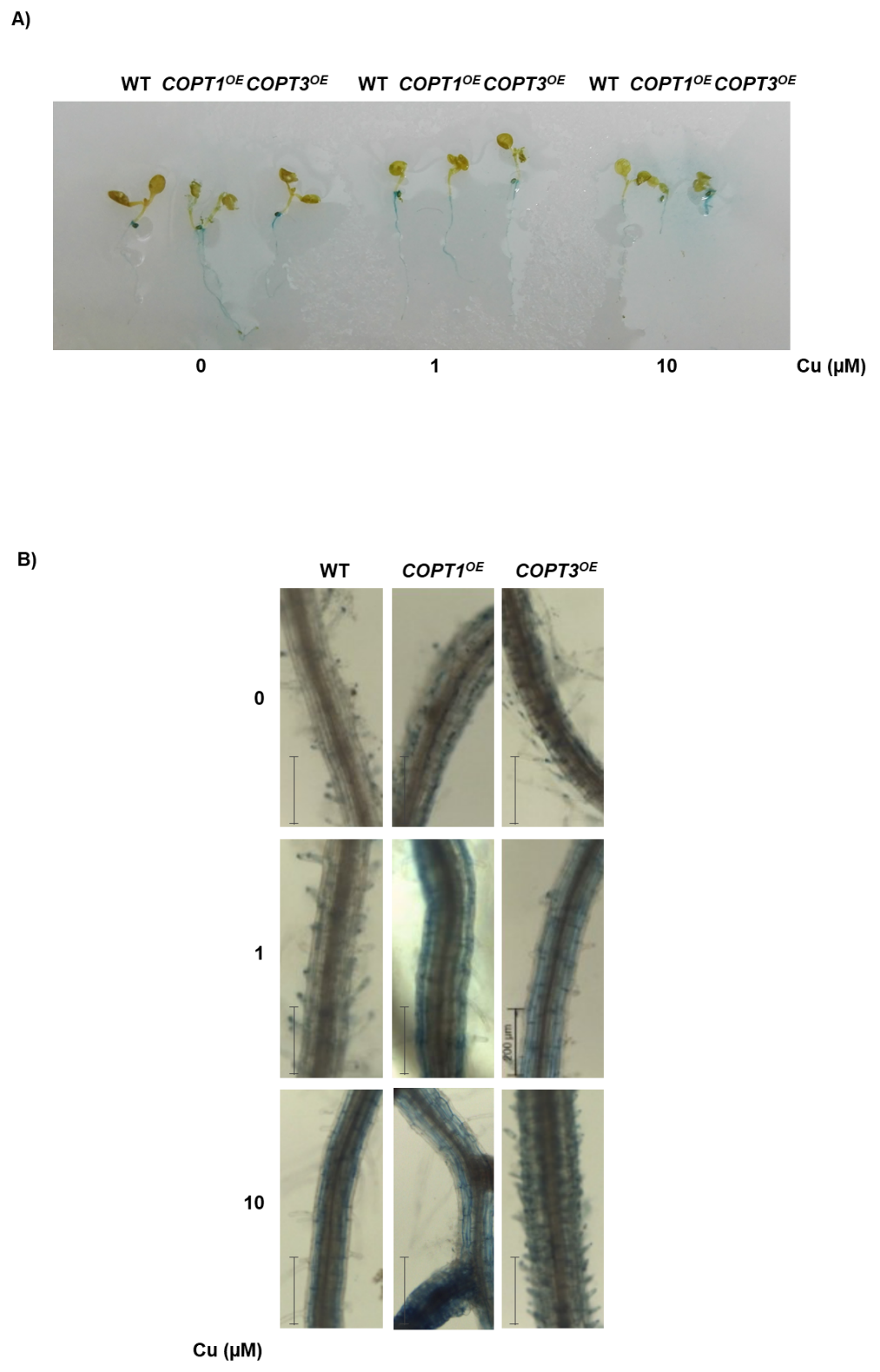


**Figure S10.** Detection of Fe^3+^ in *COPT^OE^* roots. A) Eleven-day old roots of WT, *COPT1^OE^* and *COPT3^OE^* seedlings grown under Cu deficiency (0 μM CuSO_4_), sufficiency (1 μM CuSO_4_) or Cu excess (10 μM CuSO_4_) were stained with Perl´s blue to detect Fe^3+^. Scale bar represents 1 cm. B) Detail of the roots from seedlings grown in the same conditions than in A). Scale bars represent 200 µm.

# Supplementary Tables

**Supplementary Table SI**. **Differential genes induced in *COPT1^OE^* vs. WT seedlings in low and high Cu.** MIPS code, expression values under –Cu (1/2 MS) and +Cu (1/2 MS + 10 μM Cu), gene annotation, and gene name are indicated. The differentially upregulated genes with a log2 ratio of ≥ | 1 | in the *COPT1^OE^* versus the WT seedlings in both conditions (-Cu/+Cu) under low (-Cu) and high (+Cu) Cu in the media were indicated.

| **MIPS code** | **Ratio** | **Description** | **Gene** |
| --- | --- | --- | --- |
|  | **-Cu/ +Cu** |  |  |
| At5g06170 | 3.519/ 1.806 | SUCROSE-PROTON SYMPORTER 9; Carbohydrate Transporter | SUC9 |
| At3g01185 | 3.059/ 2.005 | Similar To TAIR:AT2G21655.1 |  |
| At3g10595 | 3.049/ 1.359 | Myb Family Transcription Factor |  |
| At1g33960 | 2.804/ 2.238 | AVRRPT2-INDUCED GENE 1; GTP Binding | AIG1 |
| At1g19530 | 2.573/ 1.124 | Unknown Protein |  |
| At2g18660 | 2.507/ 2.825 | Expansin Family Protein (EXPR3) | PNP-A |
| At5g52760 | 2.294/ 1.442 | Heavy-Metal-Associated Domain-Containing Protein |  |
| At3g22231 | 2.289/ 1.657 | Pathogen And Circadian Controlled 1 | PCC1 |
| At2g04495 | 2.282/ 1.875 | Similar To TAIR:AT2G04515.1 |  |
| At2g14560 | 2.133/ 2.545 | Similar To TAIR:AT1G33840.1 | LURP1 |
| At1g01680 | 2.111/ 1.768 | U-Box Domain-Containing Protein | PUB54 |
| At5g59030 | 1.898/ 2.269 | COPPER TRANSPORTER 1; Copper Ion Transporter | COPT1 |
| At1g73800 | 1.897/ 1.523 | Calmodulin-Binding Protein |  |
| At3g47480 | 1.792/ 2.346 | Calcium-Binding EF Hand Family Protein |  |
| At1g69880 | 1.778/ 2.395 | Thioredoxin H-Type 8; Thiol-Disulfide Exchange Intermediate | ATH8 |
| At3g50770 | 1.732/ 2.220 | Calmodulin-Related Protein, Putative | CML41 |
| At5g15190 | 1.388/ 1.350 | Unknown Protein |  |
| At5g57510 | 1.384/ 1.480 | Similar To Os08g0448100 |  |
| At4g14365 | 1.354/ 1.196 | Zinc Finger (C3HC4-Type RING Finger) Family Protein | XBAT34 |
| At1g65500 | 1.302/ 2.298 | Similar To TAIR:AT1G65490.1 |  |
| At3g60540 | 1.242/ 1.247 | Sec61beta Family Protein |  |
| At5g25250 | 1.207/ 1.481 | Similar To TAIR:AT5G64870.1; TAIR:AT5G25260.1 |  |
| At3g02840 | 1.147/ 1.093 |  |  |
| At4g12490 | 1.147/ 1.492 | Protease Inhibitor/Seed Storage/Lipid Transfer Protein (LTP) Family Protein |  |
| At5g03210 | 1.140/ 1.414 | Unknown Protein |  |
| At5g02580 | 1.122/ 1.293 | Similar To TAIR:AT3G55240.1 |  |
| At4g39670 | 1.115/ 1.299 | Similar To TAIR:AT2G34690.1 |  |
| At3g28580 | 1.064/ 1.695 | AAA-Type Atpase Family Protein |  |
|  | **-Cu** |  |  |
| At2g24850 | 2.702 | TYROSINE AMINOTRANSFERASE 3; Transaminase | TAT |
| At1g53690 | 2.652 | DNA-Directed RNA Polymerases I, II, And III 7 Kda Subunit, Putative |  |
| At3g49620 | 2.587 | DARK INDUCIBLE 11; Oxidoreductase | DIN11 |
| At3g44870 | 2.330 | S-Adenosyl-L-Methionine:Carboxyl Methyltransferase Family Protein |  |
| At3g60650 | 2.219 | Unknown Protein |  |
| At2g37030 | 2.196 | Auxin-Responsive Family Protein |  |
| At3g49970 | 2.136 | Phototropic-Responsive Protein, Putative |  |
| At3g26200 | 2.073 | Cytochrome P450, Family 71, Subfamily B, Polypeptide 22); Oxygen Binding | CYP71B22 |
| At1g57630 | 1.944 | Disease Resistance Protein (TIR Class), Putative |  |
| At2g41230 | 1.934 | Similar To TAIR:AT2G44080.1 | ORS1 |
| At3g47340 | 1.896 | Asn1 (Dark Inducible 6) | ASN1 |
| At2g39030 | 1.890 | GCN5-Related N-Acetyltransferase (GNAT) Family Protein |  |
| At3g60420 | 1.855 | Similar To TAIR:AT3G60450.1 |  |
| At3g21080 | 1.834 | ABC Transporter-Related |  |
| At5g44260 | 1.829 | Zinc Finger (CCCH-Type) Family Protein |  |
| At5g14360 | 1.796 | Ubiquitin Family Protein |  |
| At2g47270 | 1.794 | Transcription Factor/ Transcription Regulator | UPB1 |
| At1g21400 | 1.782 | 2-Oxoisovalerate Dehydrogenase, Putative |  |
| At5g07100 | 1.778 | WRKY DNA-Binding Protein 26; Transcription Factor | WRKY26 |
| At5g50335 | 1.765 | Unknown Protein |  |
| At1g77380 | 1.758 | Amino Acid Permease 3; Amino Acid Permease | AAP3 |
| At4g39675 | 1.741 | Unknown Protein |  |
| At5g39160 | 1.719 | Germin-Like Protein (GLP2a) (GLP5a) |  |
| At3g01970 | 1.707 | WRKY DNA-Binding Protein 45; Transcription Factor | WRKY45 |
| At5g57760 | 1.687 |  |  |
| At5g43440 | 1.673 | 2-Oxoglutarate-Dependent Dioxygenase, Putative |  |
| At5g53980 | 1.655 | ARABIDOPSIS THALIANA HOMEOBOX PROTEIN 52; Transcription Factor | HB52 |
| At3g29370 | 1.650 | Similar To TAIR:AT5G39240.1 | P1R3 |
| At4g35770 | 1.637 | Dark Inducible 1 | SEN1 |
| At2g41280 | 1.604 | M10 | M10 |
| At3g50470 | 1.579 | Homolog of Rpw8 3 | HR3 |
| At5g22520 | 1.532 | Similar To TAIR:AT5G22530.1 |  |
| At4g28850 | 1.532 | Xyloglucan:Xyloglucosyl Transferase | XTH26 |
| At1g20520 | 1.515 | Similar To TAIR:AT1G76210.1 |  |
| At2g43530 | 1.514 | Trypsin Inhibitor, Putative |  |
| At5g06570 | 1.505 | Similar To TAIR:AT5G16080.1 |  |
| At5g49170 | 1.499 | Similar To TAIR:AT3G06840.1 |  |
| At2g26020 | 1.492 | PDF1.2b (Plant Defensin 1.2b) | PDF1.2b |
| At1g51620 | 1.490 | Protein Kinase Family Protein |  |
| At4g18250 | 1.483 | Receptor Serine/Threonine Kinase, Putative |  |
| At1g53890 | 1.483 | Similar To TAIR:AT1G53870.2 |  |
| At2g44570 | 1.481 | Glycosyl Hydrolase Family 9 Protein | GH9B12 |
| At1g30135 | 1.460 | Similar To TAIR:AT2G34600.1 | JAZ8 |
| At1g10070 | 1.451 | ATBCAT-2; Catalytic | BCAT-2 |
| At1g10340 | 1.431 | Ankyrin Repeat Family Protein |  |
| At1g53870 | 1.430 |  |  |
| At1g49860 | 1.430 | Arabidopsis Thaliana Glutathione S-Transferase (Class Phi) 14 | GSTF14 |
| At3g01830 | 1.417 | Calmodulin-Related Protein, Putative |  |
| At1g24145 | 1.410 | Unknown Protein |  |
| At3g46080 | 1.373 | Zinc Finger (C2H2 Type) Family Protein |  |
| At5g03310 | 1.360 | Auxin-Responsive Family Protein |  |
| At5g39610 | 1.357 | ANAC092/ATNAC2/ATNAC6 (Arabidopsis NAC Domain Containing Protein 92) | ANAC092 |
| At3g26740 | 1.355 | Ccr-Like | CCL |
| At4g22214 | 1.350 | Encodes A Defensin-Like (DEFL) Family Protein. |  |
| At5g44430 | 1.349 | Plant Defensin 1.2c | PDF1.2c |
| At2g22860 | 1.347 | ATPSK2 (PHYTOSULFOKINE 2 PRECURSOR); Growth Factor | PSK2 |
| At4g23700 | 1.342 | CATION/H+ EXCHANGER 17; Monovalent Cation:Proton Antiporter | CHX17 |
| At2g43870 | 1.330 | Polygalacturonase, Putative / Pectinase, Putative |  |
| At5g19890 | 1.319 | Peroxidase, Putative |  |
| At2g39400 | 1.318 | Hydrolase, Alpha/Beta Fold Family Protein |  |
| At5g13220 | 1.318 | Similar To TAIR:AT1G19180.1 | JAS1 |
| At2g17850 | 1.313 | Similar To TAIR:AT5G66170.2 |  |
| At3g46090 | 1.308 | Nucleic Acid Binding / Transcription Factor/ Zinc Ion Binding | ZAT7 |
| At2g35730 | 1.297 | Heavy-Metal-Associated Domain-Containing Protein |  |
| At5g49590 | 1.295 | Unknown Protein |  |
| At4g34810 | 1.294 | Auxin-Responsive Family Protein |  |
| At3g23150 | 1.290 | ETHYLENE RESPONSE 2; Receptor | ETR2 |
| At1g69490 | 1.286 | NAP (NAC-LIKE, ACTIVATED BY AP3/PI); Transcription Factor | ANAC029 |
| At3g55240 | 1.273 | Similar To TAIR:AT3G28990.1 |  |
| At2g33380 | 1.268 | RD20 (RESPONSIVE TO DESSICATION 20); Calcium Ion Binding | CLO3 |
| At2g05510 | 1.267 | Glycine-Rich Protein |  |
| At4g36410 | 1.264 | UBIQUITIN-CONJUGATING ENZYME 17; Ubiquitin-Protein Ligase | UBC17 |
| At4g25860 | 1.263 | Oxysterol-Binding Family Protein | ORP4A |
| At1g43160 | 1.261 | Related To AP2 6; DNA Binding / Transcription Factor | RAP2.6 |
| At1g75830 | 1.260 | PDF1.1 (Low-Molecular-Weight Cysteine-Rich 67) | LCR67 |
| At5g59070 | 1.260 | Glycosyl Transferase Family 1 Protein |  |
| At4g14130 | 1.241 | XTR7 (XYLOGLUCAN ENDOTRANSGLYCOSYLASE 7) | XTH15 |
| At1g53885 | 1.237 | Senescence-Associated Protein-Related |  |
| At2g16060 | 1.234 | Ahb1 (Arabidopsis Hemoglobin 1) | AHB1 |
| At4g28703 | 1.223 | Similar To TAIR:AT3G04300.1 |  |
| At1g16370 | 1.221 | Transporter-Related | OCT6 |
| At2g26010 | 1.215 | Plant Defensin 1.3 | PDF1.3 |
| At2g33810 | 1.212 | SQUAMOSA PROMOTER BINDING PROTEIN-LIKE 3; Transcription Factor | SPL3 |
| At3g23250 | 1.211 | Myb Domain Protein 15; DNA Binding | MYB15 |
| At1g19610 | 1.209 | PDF1.4 (Low-Molecular-Weight Cysteine-Rich 78) | LCR78 |
| At1g77120 | 1.206 | ALCOHOL DEHYDROGENASE 1; Alcohol Dehydrogenase | ADH |
| At4g32280 | 1.201 | Indoleacetic Acid-Induced Protein 29; Transcription Factor | IAA29 |
| At2g42850 | 1.198 | Cytochrome P450, Family 718; Oxygen Binding | CYP718 |
| At4g22210 | 1.182 | Low-Molecular-Weight Cysteine-Rich 85 | LCR85 |
| At3g52340 | 1.182 | SPP2; Sucrose-Phosphatase | SPP2 |
| At1g70270 | 1.180 | Contains Interpro Domain Helix-Loop-Helix DNA-Binding; (Interpro:IPR011598) |  |
| At5g63270 | 1.169 | Nitrate-Responsive NOI Protein, Putative |  |
| At1g76410 | 1.165 | Protein Binding / Zinc Ion Binding | ATL8 |
| At4g36110 | 1.164 | Auxin-Responsive Protein, Putative |  |
| At5g55450 | 1.164 | Protease Inhibitor/Seed Storage/Lipid Transfer Protein (LTP) Family Protein |  |
| At1g27670 | 1.160 | Similar To TAIR:AT1G75360.1 |  |
| At2g17660 | 1.159 | Nitrate-Responsive NOI Protein, Putative |  |
| At5g02020 | 1.152 | Similar To TAIR:AT5G59080.1 | SIS |
| At2g11810 | 1.151 | Monogalactosyldiacylglycerol Synthase Type C | MGD3 |
| At5g51390 | 1.138 | Unknown Protein |  |
| At5g02540 | 1.134 | Short-Chain Dehydrogenase/Reductase (SDR) Family Protein |  |
| At3g22460 | 1.132 | Cysteine Synthase, Putative / O-Acetylserine (Thiol)-Lyase, Putative | OASA2 |
| At2g44080 | 1.124 | Argos-Like | ARL |
| At3g51660 | 1.120 | Macrophage Migration Inhibitory Factor Family Protein / MIF Family Protein |  |
| At4g31875 | 1.112 | Contains Domain (Trans)Glycosidases (SSF51445) |  |
| At1g76210 | 1.112 | Similar To TAIR:AT1G20520.1 |  |
| At1g24575 | 1.108 | Unknown Protein |  |
| At3g57260 | 1.105 | PATHOGENESIS-RELATED PROTEIN 2; | BG2 |
| At4g26070 | 1.103 | Mitogen-Activated Protein Kinase Kinase 1; MAP Kinase Kinase/ Kinase | MEK1 |
| At4g23170 | 1.100 | EP1; Protein Kinase | CRK9 |
| At3g48970 | 1.095 | Copper-Binding Family Protein |  |
| At5g02600 | 1.094 | Heavy-Metal-Associated Domain-Containing Protein | NAKR1 |
| At5g16410 | 1.089 | Transferase Family Protein |  |
| At1g56060 | 1.088 | Similar To TAIR:AT2G32190.1 |  |
| At5g44390 | 1.077 | FAD-Binding Domain-Containing Protein |  |
| At1g76590 | 1.077 | Zinc-Binding Family Protein |  |
| At1g15040 | 1.061 | Glutamine Amidotransferase-Related |  |
| At5g28610 | 1.060 | Similar To TAIR:AT5G28630.1 |  |
| At3g60520 | 1.056 | Zinc Ion Binding |  |
| At4g11890 | 1.056 | Protein Kinase Family Protein |  |
| At4g21400 | 1.051 | Protein Kinase Family Protein | CRK28 |
| At2g29090 | 1.048 | Cytochrome P450, Family 707, Subfamily A, Polypeptide 2; Oxygen Binding | CYP707A2 |
| At1g12010 | 1.047 | 1-Aminocyclopropane-1-Carboxylate Oxidase, Putative / ACC Oxidase, Putative |  |
| At1g30757 | 1.046 | Unknown Protein |  |
| At2g22880 | 1.046 | VQ Motif-Containing Protein |  |
| At3g13435 | 1.046 | Unknown Protein |  |
| At5g46295 | 1.040 | Similar To TAIR:AT1G06475.1 |  |
| At3g55840 | 1.036 | Similar To TAIR:AT2G40000.1 |  |
| At2g48090 | 1.032 | Unknown Protein |  |
| At5g52750 | 1.030 | Heavy-Metal-Associated Domain-Containing Protein |  |
| At3g53250 | 1.027 | Auxin-Responsive Family Protein |  |
| At3g20340 | 1.027 | Similar To TAIR:AT4G21920.1 |  |
| At5g02750 | 1.027 | Zinc Finger (C3HC4-Type RING Finger) Family Protein | SGR9 |
| At2g20080 | 1.023 | Similar To TAIR:AT4G28840.1 |  |
| At5g46330 | 1.022 | FLAGELLIN-SENSITIVE 2; ATP Binding | FLS2 |
| At5g54530 | 1.021 | Similar To TAIR:AT1G61667.1 |  |
| At1g13430 | 1.020 | Sulfotransferase Family Protein | ST4C |
| At2g07739 | 1.018 | Identical To Hypothetical Mitochondrial Protein Atmg00370 |  |
| At2g40113 | 1.018 | Similar To TAIR:AT5G47635 |  |
| At4g39890 | 1.018 | Atrabh1c (Arabidopsis Rab Gtpase Homolog H1c); GTP Binding | RABH1c |
| At1g07985 | 1.013 | Similar To Expressed Protein [Oryza Sativa (Japonica Cultivar-Group)] |  |
| At5g42530 | 1.008 | Similar To TAIR:AT1G31580.1 |  |
|  | **+Cu** |  |  |
| At2g29350 | 4.705 | Senescence-Associated Gene 13; Oxidoreductase | SAG13 |
| At2g14610 | 4.633 | Pathogenesis-Related Gene 1 | PR1 |
| At3g60140 | 3.815 | DIN2 (DARK INDUCIBLE 2); Hydrolase, Hydrolyzing O-Glycosyl Compounds | BGLU30 |
| At1g61800 | 3.371 | Glucose-6-Phosphate/Phosphate Translocator 2 | GPT2 |
| At1g47395 | 3.318 | Similar To TAIR:AT1G47400.1 |  |
| At2g38340 | 3.248 | AP2 Domain-Containing Transcription Factor, Putative (DRE2B) | DREB19 |
| At5g26220 | 3.189 | Chac-Like Family Protein |  |
| At5g13210 | 3.166 | Similar To TAIR:AT3G24780.1; TAIR:AT5G43400.1; TAIR:AT5G43390.1 |  |
| At2g44460 | 3.037 | Glycosyl Hydrolase Family 1 Protein | BGLU28 |
| At1g23730 | 3.018 | Carbonic Anhydrase, Putative / Carbonate Dehydratase, Putative | BCA3 |
| At4g12735 | 2.971 | Unknown Protein |  |
| At3g49580 | 2.950 | Similar To TAIR:AT3G49570.1 | LSU1 |
| At2g38823 | 2.921 | Similar To TAIR:AT3G54520.1 |  |
| At2g41240 | 2.914 | Basic Helix-Loop-Helix (Bhlh) Family Protein | BHLH100 |
| At2g04070 | 2.825 | MATE Efflux Family Protein |  |
| At1g12030 | 2.740 | Similar To TAIR:AT1G62420.1 |  |
| At5g17220 | 2.659 | GLUTATHIONE S-TRANSFERASE 26; Glutathione Transferase | GSTF12 |
| At5g48850 | 2.657 | Male Sterility MS5 Family Protein | SDI1 |
| At2g20800 | 2.634 | NAD(P)H DEHYDROGENASE B4; NADH Dehydrogenase | NDB4 |
| At2g18190 | 2.612 | AAA-Type Atpase Family Protein |  |
| At2g21640 | 2.584 | Similar To TAIR:AT3G05570.1; TAIR:AT4G39235.1 |  |
| At2g14620 | 2.577 | Xyloglucan:Xyloglucosyl Transferase, Putative | XTH10 |
| At4g04490 | 2.531 | Protein Kinase Family Protein | CRK36 |
| At3g08860 | 2.528 | Alanine--Glyoxylate Aminotransferase, Putative | PYD4 |
| At2g04050 | 2.525 | MATE Efflux Family Protein |  |
| At3g56980 | 2.523 | ORG3 (OBP3-Responsive Gene 3); DNA Binding / Transcription Factor | BHLH039 |
| At1g05680 | 2.510 | UDP-Glucoronosyl/UDP-Glucosyl Transferase Family Protein | UGT74E2 |
| At1g47400 | 2.466 | Similar To TAIR:AT1G47395.1 |  |
| At3g22370 | 2.463 | Alternative Oxidase 1A; Alternative Oxidase | AOX1A |
| At2g41730 | 2.456 | Similar To TAIR:AT5G24640.1 |  |
| At3g56970 | 2.453 | ORG2 (OBP3-Responsive Gene 2); DNA Binding / Transcription Factor | BHLH038 |
| At2g18193 | 2.436 | AAA-Type Atpase Family Protein |  |
| At3g25180 | 2.427 | Cytochrome P450, Family 82, Subfamily G, Polypeptide 1; Oxygen Binding | CYP82G1 |
| At3g13080 | 2.417 | ATMRP3 (Arabidopsis Thaliana Multidrug Resistance-Associated Protein 3) | ABCC3 |
| At1g17180 | 2.407 | Arabidopsis Thaliana Glutathione S-Transferase (Class Tau) 25 | GSTU25 |
| At5g42800 | 2.355 | DIHYDROFLAVONOL 4-REDUCTASE; Dihydrokaempferol 4-Reductase | DFR |
| At5g24200 | 2.349 | Triacylglycerol Lipase |  |
| At3g50610 | 2.334 | Similar To TAIR:AT1G59835.1 |  |
| At3g13950 | 2.292 | Similar To TAIR:AT4G13266.1 (GB:ABE80572.1) |  |
| At5g62480 | 2.273 | GLUTATHIONE S-TRANSFERASE TAU 9; Glutathione Transferase | GSTU9 |
| At2g26560 | 2.240 | PLP2 (PHOSPHOLIPASE A 2A); Nutrient Reservoir | PLA IIA |
| At1g43910 | 2.205 | Protein Phosphatase 2C, Putative / PP2C, Putative |  |
| At1g02470 | 2.202 | Similar To TAIR:AT1G02475.1 |  |
| At5g24640 | 2.191 | Similar To TAIR:AT2G41730.1 |  |
| At4g21680 | 2.177 | Proton-Dependent Oligopeptide Transport (POT) Family Protein | NRT1.8 |
| At2g36790 | 2.175 | UDP-Glucosyl Transferase 73C6; UDP-Glucosyltransferase | UGT73C6 |
| At3g05400 | 2.167 | Sugar Transporter, Putative |  |
| At5g04150 | 2.152 | Basic Helix-Loop-Helix (Bhlh) Family Protein | BHLH101 |
| At1g21520 | 2.149 | Unknown Protein |  |
| At4g37370 | 2.145 | Cytochrome P450, Family 81, Subfamily D, Polypeptide 8; Oxygen Binding | CYP81D8 |
| At3g01600 | 2.136 | Arabidopsis NAC Domain Containing Protein 44; Transcription Factor | anac044 |
| At4g32810 | 2.106 | Carotenoid Cleavage Dioxygenase 8 | CCD8 |
| At3g29250 | 2.101 | Oxidoreductase |  |
| At5g22460 | 2.092 | Esterase/Lipase/Thioesterase Family Protein |  |
| At1g32350 | 2.065 | ALTERNATIVE OXIDASE 1D; Alternative Oxidase | AOX1D |
| At2g36800 | 2.055 | DON-GLUCOSYLTRANSFERASE; UDP-Glycosyltransferase | DOGT1 |
| At3g29970 | 2.033 | Germination Protein-Related |  |
| At3g59450 | 2.029 | Calcium-Binding EF Hand Family Protein |  |
| At1g68765 | 2.019 | Inflorescence Deficient In Abscission | IDA |
| At1g52315 | 2.003 | Similar To TAIR:AT1G79910.1 |  |
| At2g47190 | 1.989 | Myb Domain Protein 2; DNA Binding / Transcription Factor | MYB2 |
| At3g04070 | 1.987 | Arabidopsis NAC Domain Containing Protein 47; Transcription Factor | anac047 |
| At2g02990 | 1.940 | RIBONUCLEASE 1; Endoribonuclease | ATRNS1 |
| At5g18270 | 1.921 |  | ANAC087 |
| At2g08986 | 1.911 |  |  |
| At3g24780 | 1.893 | Similar To TAIR:AT5G13210.1; TAIR:AT5G43400.1; TAIR:AT5G43390.1 |  |
| At5g53450 | 1.893 | OBP3-RESPONSIVE GENE 1; Kinase | ORG1 |
| At2g36780 | 1.893 | UDP-Glucoronosyl/UDP-Glucosyl Transferase Family Protein |  |
| At2g03230 | 1.889 | Similar To TAIR:AT1G14060.1 |  |
| At1g66700 | 1.889 | S-Adenosyl-L-Methionine:Carboxyl Methyltransferase Family Protein | PXMT1 |
| At4g05370 | 1.876 | Similar To TAIR:AT1G43910.1 |  |
| At4g05380 | 1.875 | AAA-Type Atpase Family Protein |  |
| At2g03760 | 1.867 | ST (Steroid Sulfotransferase); Sulfotransferase | SOT1 |
| At3g03270 | 1.867 | Universal Stress Protein (USP) Family Protein |  |
| At5g27760 | 1.857 | Hypoxia-Responsive Family Protein |  |
| At4g23990 | 1.845 | Cellulose Synthase-Like G3; Transferase | CSLG3 |
| At5g07550 | 1.837 | GRP19 (Glycine Rich Protein 19) | GRP19 |
| At5g33290 | 1.820 | XYLOGALACTURONAN DEFICIENT 1; Catalytic | XGD1 |
| At3g21720 | 1.812 | Isocitrate Lyase, Putative | ICL |
| At4g36700 | 1.798 | Cupin Family Protein |  |
| At2g30770 | 1.795 | Cytochrome P450, Family 71, Subfamily A, Polypeptide 13; Oxygen Binding | CYP71A13 |
| At5g40770 | 1.785 | Prohibitin 3 | PHB3 |
| At3g49570 | 1.785 | Similar To TAIR:AT5G24660.1 | LSU3 |
| At1g74010 | 1.779 | Strictosidine Synthase Family Protein |  |
| At3g13130 | 1.767 | Similar To TAIR:AT2G04515.1 |  |
| At5g43450 | 1.766 | 2-Oxoglutarate-Dependent Dioxygenase, Putative |  |
| At2g36750 | 1.757 | UGT72C1 (UDP-Glucosyl Transferase 72C1) | UGT73C1 |
| At4g37290 | 1.746 | Similar To TAIR:AT2G23270.1 |  |
| At5g54560 | 1.743 | Similar To TAIR:AT5G54550.1 |  |
| At2g35480 | 1.742 | Similar To TAIR:AT1G32260.1 |  |
| At2g25625 | 1.739 | Similar To Os05g0575000 Group |  |
| At4g08555 | 1.738 | Unknown Protein |  |
| At5g07610 | 1.731 | F-Box Family Protein |  |
| At5g07600 | 1.730 | Oleosin / Glycine-Rich Protein |  |
| At3g56730 | 1.727 | Similar To TAIR:AT3G62050.1 |  |
| At4g22870 | 1.725 | Leucoanthocyanidin Dioxygenase, Putative / Anthocyanidin Synthase, Putative |  |
| At3g45730 | 1.724 | Unknown Protein |  |
| At1g24580 | 1.719 | Zinc Finger (C3HC4-Type RING Finger) Family Protein |  |
| At4g37990 | 1.711 | Eli3-2 (Elicitor-Activated Gene 3) | CAD8 |
| At3g28210 | 1.703 | Zinc Ion Binding | PMZ |
| At3g46070 | 1.692 | Zinc Finger (C2H2 Type) Family Protein |  |
| At3g21500 | 1.689 | 1-Deoxy-D-Xylulose-5-Phosphate Synthase | DXPS1 |
| At2g47780 | 1.689 | Rubber Elongation Factor (REF) Protein-Related |  |
| At5g57730 | 1.682 | Unknown Protein |  |
| At2g40340 | 1.676 | AP2 Domain-Containing Transcription Factor, Putative (DRE2B) | ERF48 |
| At1g29640 | 1.674 | Similar To TAIR:AT2G34340.1 |  |
| At2g18600 | 1.672 | RUB1-Conjugating Enzyme, Putative |  |
| At3g25655 | 1.669 | Unknown Protein | IDL1 |
| At5g24660 | 1.664 | Similar To TAIR:AT5G24655.1 | LSU2 |
| At3g58270 | 1.659 | Meprin And TRAF Homology Domain-Containing Protein |  |
| At5g06190 | 1.659 | Similar To TAIR:AT3G58540.1 |  |
| At4g05020 | 1.658 | NAD(P)H DEHYDROGENASE B2; Disulfide Oxidoreductase | NDB2 |
| At2g26400 | 1.658 | ARD/ATARD3 (ACIREDUCTONE DIOXYGENASE) | ARD |
| At1g54575 | 1.651 | Unknown Protein |  |
| At4g35180 | 1.650 | LYS/HIS TRANSPORTER 7; Amino Acid Permease | LHT7 |
| At2g27840 | 1.649 | HDT4 (Histone Deacetylase 13) | HDA13 |
| At4g05136 | 1.641 | Similar To TAIR:AT3G52410.1 |  |
| At1g56650 | 1.635 | PAP1 (PRODUCTION OF ANTHOCYANIN PIGMENT 1); | MYB75 |
| At3g55620 | 1.634 | EMB1624 (EMBRYO DEFECTIVE 1624); Translation Initiation Factor | eIF6A |
| At5g37600 | 1.629 | ATGSR1 (Arabidopsis Thaliana Glutamine Synthase Clone R1) | GLN1;1 |
| At3g57380 | 1.629 | Similar To TAIR:AT2G41640.1 |  |
| At5g67510 | 1.623 | 60S Ribosomal Protein L26 (RPL26B) |  |
| At2g14247 | 1.620 | Unknown Protein |  |
| At1g26390 | 1.616 | FAD-Binding Domain-Containing Protein |  |
| At2g03770 | 1.612 | Sulfotransferase Family Protein |  |
| At2g43510 | 1.596 | Arabidopsis Thaliana Trypsin Inhibitor Protein 1 | ATTI1 |
| At1g14720 | 1.595 | XTR2 (XYLOGLUCAN ENDOTRANSGLYCOSYLASE RELATED 2); | XTH28 |
| At1g32870 | 1.593 | Arabidopsis NAC Domain Containing Protein 13; Transcription Factor | ANAC013 |
| At2g04460 | 1.590 | Similar To TAIR:AT2G10090.1 |  |
| At2g07981 | 1.590 | Similar To TAIR:AT2G08986.1 |  |
| At3g23120 | 1.589 | Leucine-Rich Repeat Family Protein | RLP38 |
| At3g11580 | 1.589 | DNA-Binding Protein, Putative |  |
| At3g54150 | 1.585 | Embryo-Abundant Protein-Related |  |
| At1g73805 | 1.583 | Calmodulin-Binding Protein | SARD1 |
| At2g23270 | 1.564 | Similar To TAIR:AT4G37290.1 |  |
| At2g35070 | 1.560 | Similar To TAIR:AT2G35090.1 |  |
| At3g63380 | 1.560 | Calcium-Transporting Atpase, Plasma Membrane-Type, Putative |  |
| At4g37570 | 1.556 | Similar To TAIR:AT3G52410.1 |  |
| At1g69930 | 1.549 | Arabidopsis Thaliana Glutathione S-Transferase (Class Tau) 11 | GSTU11 |
| At5g53420 | 1.547 | Similar To TAIR:AT4G27900.2 |  |
| At1g26400 | 1.530 | FAD-Binding Domain-Containing Protein |  |
| At2g43570 | 1.527 | Chitinase, Putative | CHI |
| At5g39520 | 1.513 | Similar To TAIR:AT5G39530.1 |  |
| At1g52855 | 1.510 | Similar To TAIR:AT2G20835.1 |  |
| At2g36770 | 1.509 | UDP-Glucoronosyl/UDP-Glucosyl Transferase Family Protein |  |
| At5g39850 | 1.504 | 40S Ribosomal Protein S9 (RPS9C) |  |
| At3g52410 | 1.494 | Similar To TAIR:AT5G36010.1 |  |
| At1g31760 | 1.473 | SWIB Complex BAF60b Domain-Containing Protein |  |
| At5g54100 | 1.465 | Band 7 Family Protein |  |
| At3g04410 | 1.463 | Transcription Factor |  |
| At4g21215 | 1.456 | Similar To Hypothetical Protein Mtrdraft_AC123975g8v1 [Medicago Truncatula] |  |
| At5g47880 | 1.456 | EUKARYOTIC RELEASE FACTOR 1-1) Translation Release Factor | ERF1-1 |
| At2g20720 | 1.452 | Pentatricopeptide (PPR) Repeat-Containing Protein |  |
| At3g57440 | 1.446 | Unknown Protein |  |
| At4g22880 | 1.445 | Ldox (Tannin Deficient Seed 4) | ANS |
| At3g27060 | 1.443 | TSO2 (TSO2); Ribonucleoside-Diphosphate Reductase | TSO2 |
| At1g26380 | 1.441 | FAD-Binding Domain-Containing Protein |  |
| At3g46280 | 1.436 | Protein Kinase-Related |  |
| At5g60520 | 1.433 | EDA38 (Embryo Sac Development Arrest 38); Selenium Binding |  |
| At1g67760 | 1.427 | ATP Binding / Protein Binding / Unfolded Protein Binding |  |
| At5g40690 | 1.418 | Similar To Unknown Protein [Arabidopsis Thaliana] (TAIR:AT2G41730.1) |  |
| At1g19210 | 1.418 | AP2 Domain-Containing Transcription Factor, Putative |  |
| At2g28820 | 1.416 | Structural Constituent Of Ribosome |  |
| At2g43660 | 1.416 | Glycosyl Hydrolase Family Protein 17 |  |
| At2g43660 | 1.416 | Glycosyl Hydrolase Family Protein 17 |  |
| At4g17670 | 1.411 | Senescence-Associated Protein-Related |  |
| At5g17760 | 1.411 | AAA-Type Atpase Family Protein |  |
| At4g01610 | 1.408 | Cathepsin B-Like Cysteine Protease, Putative |  |
| At4g01610 | 1.408 | Cathepsin B-Like Cysteine Protease, Putative |  |
| At3g28010 | 1.400 | Similar To TAIR:AT5G38037.1 |  |
| At2g24762 | 1.397 | Similar To TAIR:AT4G31730.1 | GDU4 |
| At1g17960 | 1.395 | Threonyl-Trna Synthetase, Putative / Threonine--Trna Ligase, Putative |  |
| At3g06600 | 1.393 | Unknown Protein |  |
| At3g08590 | 1.391 | 2,3-Biphosphoglycerate-Independent Phosphoglycerate Mutase, Putative |  |
| At1g52890 | 1.389 | Arabidopsis NAC Domain Containing Protein 19; Transcription Factor | ANAC019 |
| At1g29290 | 1.387 | Similar To Hypothetical Protein Mtrdraft_AC152407g2v1 |  |
| At3g44300 | 1.386 | Nitrilase 2 | NIT2 |
| At4g09600 | 1.385 | Gast1 Protein Homolog 3 | GASA3 |
| At3g51860 | 1.384 | Cation Exchanger 3; Cation:Cation Antiporter | CAX3 |
| At3g61630 | 1.375 | CYTOKININ RESPONSE FACTOR 6; DNA Binding / Transcription Factor | CRF6 |
| At3g04320 | 1.368 | Endopeptidase Inhibitor |  |
| At3g25250 | 1.361 | OXIDATIVE SIGNAL-INDUCIBLE1; Kinase | AGC2 |
| At5g27420 | 1.359 | Zinc Finger (C3HC4-Type RING Finger) Family Protein | ATL31 |
| At3g18290 | 1.358 | Zinc Finger C3HC4-Type RING Finger) Family Protein | BTS |
| At3g27630 | 1.354 | Similar To TAIR:AT5G40460.1 |  |
| At2g16700 | 1.352 | ACTIN DEPOLYMERIZING FACTOR 5; Actin Binding | ADF5 |
| At1g76960 | 1.348 | Unknown Protein |  |
| At3g28510 | 1.347 | AAA-Type Atpase Family Protein |  |
| At3g16565 | 1.342 | ATP Binding / Alanine-Trna Ligase |  |
| At3g25882 | 1.341 | Nim1-Interacting 2 | NIMIN-2 |
| At5g59220 | 1.341 | Protein Phosphatase 2C, Putative / PP2C, Putative | HAI1 |
| At3g30160 | 1.340 | Similar To TAIR:AT1G35320.1 |  |
| At5g24980 | 1.337 | Similar To TAIR:AT5G10745.1 |  |
| At1g26420 | 1.328 | FAD-Binding Domain-Containing Protein |  |
| At5g14730 | 1.322 | Similar To TAIR:AT3G01513.1 |  |
| At5g02780 | 1.302 | In2-1 Protein, Putative | GSTL1 |
| At1g64490 | 1.296 | Similar To TAIR:AT5G42060.1 |  |
| At3g26830 | 1.292 | PAD3 (PHYTOALEXIN DEFICIENT 3); Oxygen Binding | CYP71B15 |
| MIR395e | 1.292 |  |  |
| At3g04420 | 1.292 | Arabidopsis NAC Domain Containing Protein 48; Transcription Factor | anac048 |
| At1g60730 | 1.287 | Aldo/Keto Reductase Family Protein |  |
| At2g23110 | 1.282 | Similar To TAIR:AT2G23120.1 |  |
| At4g26460 | 1.280 | Similar To GAMT1, S-Adenosylmethionine-Dependent Methyltransferase/ |  |
| At2g44070 | 1.280 | Eukaryotic Translation Initiation Factor 2B Family Protein / Eif-2B Family Protein |  |
| At2g47010 | 1.275 | Similar To TAIR:AT1G17030.1 |  |
| At1g01810 | 1.272 | Unknown Protein |  |
| At1g77655 | 1.258 | Unknown Protein |  |
| At1g02750 | 1.257 | Similar To TAIR:AT4G02200.1 |  |
| At2g14580 | 1.255 | Arabidopsis Thaliana Basic Pathogenesis-Related Protein 1 | PRB1 |
| At1g54530 | 1.252 | Calcium-Binding EF Hand Family Protein |  |
| At2g43610 | 1.245 | Glycoside Hydrolase Family 19 Protein |  |
| At1g07900 | 1.240 | LOB Domain Protein 1 / Lateral Organ Boundaries Domain Protein 1 | LBD1 |
| At5g11780 | 1.238 | Similar To GB:BAD53434.1 |  |
| At1g23020 | 1.237 | FERRIC REDUCTION OXIDASE 3; Ferric-Chelate Reductase | FRO3 |
| At5g03860 | 1.235 | Malate Synthase, Putative | MLS |
| At3g44040 | 1.234 | MATE Efflux Protein-Related |  |
| At4g09210 | 1.234 | Similar To TAIR:AT4G09260.1 |  |
| At5g22800 | 1.229 | Aminoacyl-Trna Synthetase Family Protein | EMB1030 |
| At3g44350 | 1.227 | Arabidopsis NAC Domain Containing Protein 61; Transcription Factor | anac061 |
| At1g65370 | 1.225 | Meprin And TRAF Homology Domain-Containing Protein |  |
| At5g61160 | 1.223 | ANTHOCYANIN 5-AROMATIC ACYLTRANSFERASE 1; Transferase | AACT1 |
| At3g18250 | 1.223 | Unknown Protein |  |
| At1g02920 | 1.214 | ATGSTF7 (GLUTATHIONE S-TRANSFERASE 11); Glutathione Transferase | GST11 |
| At3g23605 | 1.213 | UBX Domain-Containing Protein |  |
| At1g27020 | 1.208 | Similar To TAIR:AT1G27030.1 |  |
| At3g10930 | 1.207 | Unknown Protein |  |
| At1g02220 | 1.206 | Arabidopsis NAC Domain Containing Protein 3; Transcription Factor | ANAC003 |
| At3g44010 | 1.203 | 40S Ribosomal Protein S29 (RPS29B) |  |
| At1g36640 | 1.201 | Similar To TAIR:AT1G36622.1 |  |
| At1g51850 | 1.199 | Leucine-Rich Repeat Protein Kinase, Putative |  |
| At1g13330 | 1.192 | Similar To TAIR:AT3G28770.1 | AHP2 |
| At2g02850 | 1.190 | ARPN (PLANTACYANIN); Copper Ion Binding | ARPN |
| At1g66380 | 1.189 | Myb Domain Protein 114; DNA Binding / Transcription Factor | MYB114 |
| At1g23550 | 1.184 | SIMILAR TO RCD ONE 2; NAD+ ADP-Ribosyltransferase | SRO2 |
| At5g13320 | 1.180 | Pbs3 (Avrpphb Susceptible 3) | GDG1 |
| At3g47965 | 1.174 | Unknown Protein |  |
| At3g18610 | 1.172 | ATRANGAP1 (RAN GTPASE-ACTIVATING PROTEIN 1); Nucleic Acid Binding | NUC-L2 |
| At4g09260 | 1.170 | Similar To TAIR:AT4G09210.1 |  |
| At4g01430 | 1.166 | Nodulin Mtn21 Family Protein |  |
| At4g01430 | 1.166 | Nodulin Mtn21 Family Protein |  |
| At1g61580 | 1.165 | ARABIDOPSIS RIBOSOMAL PROTEIN 2; Structural Constituent Of Ribosome | ARP2 |
| At1g61930 | 1.161 | Similar To TAIR:AT1G11700.1 |  |
| At1g52905 | 1.161 | Similar To TAIR:AT1G27565.1 |  |
| At5g20160 | 1.156 | Ribosomal Protein L7Ae/L30e/S12e/Gadd45 Family Protein |  |
| At5g14930 | 1.155 | Senescence-Associated Gene 101 | SAG101 |
| At1g26470 | 1.153 | Similar To Os05g0512500 |  |
| At5g05250 | 1.153 | Similar To TAIR:AT3G56360.1 |  |
| At2g03130 | 1.148 | Ribosomal Protein L12 Family Protein |  |
| At2g42660 | 1.147 | Myb Family Transcription Factor |  |
| At2g32220 | 1.136 | 60S Ribosomal Protein L27 (RPL27A) |  |
| At1g61450 | 1.132 | Similar To Os05g0275600 |  |
| At3g57860 | 1.127 | (UV-B-INSENSITIVE 4-LIKE); Unknown Protein | OSD1 |
| At4g15910 | 1.126 | ATDI21 (Arabidopsis Thaliana Drought-Induced 21) | DI21 |
| At1g76470 | 1.125 | Cinnamoyl-Coa Reductase |  |
| At3g14060 | 1.125 | Similar To TAIR:AT1G54120.1 |  |
| At3g07590 | 1.118 | Small Nuclear Ribonucleoprotein D1, Putative / Snrnp Core Protein D1 |  |
| At3g10110 | 1.117 | Maternal Effect Embryo Arrest 67; Protein Translocase | MEE67 |
| At4g04620 | 1.116 | AUTOPHAGY 8B; Microtubule Binding | ATG8B |
| At5g11100 | 1.114 | C2 Domain-Containing Protein | SYTD |
| At2g14460 | 1.111 | Unknown Protein |  |
| At5g42830 | 1.108 | Transferase Family Protein |  |
| At5g53690 | 1.107 | Unknown Protein |  |
| At5g01540 | 1.107 | Lectin Protein Kinase, Putative | LECRKA4.1 |
| At3g57490 | 1.106 | 40S Ribosomal Protein S2 (RPS2D) |  |
| At5g09270 | 1.102 | Similar To Conserved Hypothetical Protein [Medicago Truncatula] |  |
| At3g44320 | 1.101 | Nit3 (Nitrilase 3) | NIT3 |
| At5g58570 | 1.097 |  |  |
| At2g42310 | 1.096 | Similar To TAIR:AT3G57785.1); |  |
| At4g38100 | 1.095 | Threonine Endopeptidase |  |
| At2g30140 | 1.089 | UDP-Glucoronosyl/UDP-Glucosyl Transferase Family Protein |  |
| At5g64810 | 1.088 | WRKY DNA-Binding Protein 51; Transcription Factor | WRKY51 |
| At3g05937 | 1.087 | Similar To TAIR:AT5G26731.1 |  |
| At2g44840 | 1.086 | ETHYLENE-RESPONSIVE ELEMENT BINDING FACTOR 13 | ERF13 |
| At4g02380 | 1.085 | Sag21 (Senescence-Associated Gene 21) | LEA5 |
| At3g03470 | 1.084 | Cytochrome P450, Family 87, Subfamily A, Polypeptide 9; Oxygen Binding | CYP89A9 |
| At2g17040 | 1.076 | Arabidopsis NAC Domain Containing Protein 36; Transcription Factor | anac036 |
| At1g30475 | 1.075 | Similar To TAIR:AT1G56200.1 |  |
| At1g69790 | 1.075 | Protein Kinase, Putative |  |
| At3g61990 | 1.072 | O-Methyltransferase Family 3 Protein | OMTF3 |
| At2g42790 | 1.068 | CITRATE SYNTHASE 3; Citrate (SI)-Synthase | CSY3 |
| At5g62960 | 1.067 | Similar To TAIR:AT1G10660.1 |  |
| At4g14310 | 1.066 | Similar To TAIR:AT2G22795.1 |  |
| At5g57720 | 1.063 | Transcriptional Factor B3 Family Protein |  |
| At5g37660 | 1.062 | Receptor-Like Protein Kinase-Related | PDLP7 |
| At1g68200 | 1.060 | Zinc Finger (CCCH-Type) Family Protein |  |
| At3g55890 | 1.059 | Yippee Family Protein |  |
| At5g13080 | 1.051 | WRKY DNA-Binding Protein 75; Transcription Factor | WRKY75 |
| At1g49910 | 1.048 | WD-40 Repeat Family Protein / Mitotic Checkpoint Protein, Putative | BUB3.2 |
| At1g44130 | 1.048 | Nucellin Protein, Putative |  |
| At4g28460 | 1.045 | Unknown Protein |  |
| At2g33793 | 1.044 | Similar To TAIR:AT2G46980.2 |  |
| At1g09815 | 1.043 | DNA Polymerase Delta Subunit 4 Family | POLD4 |
| At1g56045 | 1.041 | Ribosomal Protein L41 Family Protein |  |
| At3g43680 | 1.041 | Similar To TAIR:AT3G60930.1 |  |
| At2g21870 | 1.039 | Identical To Probable ATP Synthase 24 Kda Subunit, | MGP1 |
| At1g20350 | 1.039 | Arabidopsis Thaliana Translocase Inner Membrane Subunit 17-1 | TIM17-1 |
| At4g28450 | 1.035 | Transducin Family Protein / WD-40 Repeat Family Protein |  |
| At4g20000 | 1.035 | VQ Motif-Containing Protein |  |
| At1g73965 | 1.034 | CLAVATA3/ESR-RELATED 13; Receptor Binding | CLE13 |
| At4g37870 | 1.031 | ATP Binding / Phosphoenolpyruvate Carboxykinase (ATP) | PCK1 |
| At3g50910 | 1.030 | Similar To TAIR:AT5G66480.1 |  |
| At4g31290 | 1.028 | Chac-Like Family Protein |  |
| At2g29490 | 1.021 | GLUTATHIONE S-TRANSFERASE 19; Glutathione Transferase | GSTU1 |
| At4g02390 | 1.017 | ARABIDOPSIS POLY(ADP-RIBOSE) POLYMERASE | APP |
| At1g77840 | 1.016 | Eukaryotic Translation Initiation Factor 5, Putative / Eif-5, Putative |  |
| At3g09020 | 1.016 | Alpha 1,4-Glycosyltransferase Family Protein |  |
| At3g53040 | 1.014 | Late Embryogenesis Abundant Protein, Putative / LEA Protein, Putative |  |
| At2g29460 | 1.012 | GLUTATHIONE S-TRANSFERASE 22; Glutathione Transferase | GSTU4 |
| At4g26200 | 1.012 | 1-Amino-Cyclopropane-1-Carboxylate Synthase 7 | ACS7 |
| At2g45490 | 1.003 | ATAURORA3; ATP Binding / Histone Serine Kinase(H3-S10 Specific) / | AUR3 |
| At5g19300 | 1.001 | Similar To Os04g0244500 |  |

**Supplementary Table SII**. **Differential genes repressed in *COPT1^OE^* vs. WT seedlings in low and high Cu.** MIPS code, expression values under –Cu (1/2 MS) and +Cu (1/2 MS + 10 μM Cu), gene annotation, and gene name are indicated. The differentially downregulated genes with a log2 ratio of ≥ | 1 | in the *COPT1^OE^* versus the WT seedlings in both conditions (-Cu/+Cu) under low (-Cu) and high (+Cu) Cu in the media were indicated.

| **MIPS code** | **Ratio** | **Description** | **Gene** |
| --- | --- | --- | --- |
|  | **-Cu/ +Cu** |  |  |
| At4g19690 | -1.736/ -1.775 | Iron-Regulated Transporter 1 | IRT1 |
| At3g28270 | -1.621/ -1.782 | Similar To TAIR:AT3G28290.1; TAIR:AT3G28300.1 |  |
|  | **-Cu** |  |  |
| At2g27420 | -2.442 | Cysteine Proteinase, Putative |  |
| At1g69070 | -1.872 | Similar To TAIR:AT3G28770.1 |  |
| At3g28345 | -1.819 | ABC Transporter Family Protein | ABCB15 |
| At4g17090 | -1.727 | CT-BMY (BETA-AMYLASE 8); Beta-Amylase | BAM3 |
| At2g42540 | -1.644 | Cor15a (Cold-Regulated 15a) | COR15 |
| At5g03090 | -1.618 | Similar To TAIR:AT1G53480.1 |  |
| At3g58810 | -1.585 | MTPA2; Efflux Permease/ Zinc Ion Transporter | MTP3 |
| At1g13650 | -1.373 | Similar To TAIR:AT2G03810.3 |  |
| At1g05680 | -1.266 | UDP-Glucoronosyl/UDP-Glucosyl Transferase Family Protein | UGT74E2 |
| At1g29660 | -1.257 | GDSL-Motif Lipase/Hydrolase Family Protein |  |
| At5g13170 | -1.228 | Nodulin Mtn3 Family Protein | SWEET15 |
| At3g21720 | -1.204 | Isocitrate Lyase, Putative | ICL |
| At2g28290 | -1.198 | Syd (Splayed) | CHR3 |
| At2g23000 | -1.150 | SCPL10 (Serine Carboxypeptidase-Like 10) | scpl10 |
| At1g54040 | -1.082 | Epithiospecifier Protein | ESP |
| At1g69200 | -1.033 | Pfkb-Type Carbohydrate Kinase Family Protein | FLN2 |
| At3g01550 | -1.018 | Triose Phosphate/Phosphate Translocator, Putative | PPT2 |
| At5g35970 | -1.004 | DNA-Binding Protein, Putative |  |
|  | **+Cu** |  |  |
| [At4g26530](http://genome-www4.stanford.edu/cgi-bin/SMD/source/sourceResult?choice=Gene&option=Name&criteria=At4g26530) | -3.344 | Fructose-Bisphosphate Aldolase |  |
| [At4g31940](http://genome-www4.stanford.edu/cgi-bin/SMD/source/sourceResult?choice=Gene&option=Name&criteria=At4g31940) | -2.586 | Cytochrome P450, Family 82, Subfamily C, Polypeptide 4 | CYP82C4 |
| [At5g51720](http://genome-www4.stanford.edu/cgi-bin/SMD/source/sourceResult?choice=Gene&option=Name&criteria=At5g51720) | -2.306 | Similar To Zinc Finger, CDGSH-Type Domain 2 |  |
| [At4g37410](http://genome-www4.stanford.edu/cgi-bin/SMD/source/sourceResult?choice=Gene&option=Name&criteria=At4g37410) | -2.179 | Cytochrome P450, Family 81, Subfamily F, Polypeptide 4 | CYP81F4 |
| [At1g66100](http://genome-www4.stanford.edu/cgi-bin/SMD/source/sourceResult?choice=Gene&option=Name&criteria=At1g66100) | -2.171 | Thionin, Putative |  |
| [At5g01600](http://genome-www4.stanford.edu/cgi-bin/SMD/source/sourceResult?choice=Gene&option=Name&criteria=At5g01600) | -2.166 | Ferretin 1; Ferric Iron Binding | FER1 |
| [At2g01520](http://genome-www4.stanford.edu/cgi-bin/SMD/source/sourceResult?choice=Gene&option=Name&criteria=At2g01520) | -2.153 | Major Latex Protein-Related / MLP-Related | MLP328 |
| [At3g25820](http://genome-www4.stanford.edu/cgi-bin/SMD/source/sourceResult?choice=Gene&option=Name&criteria=At3g25820) | -2.071 | #N/A | TPS-CIN |
| [At4g11320](http://genome-www4.stanford.edu/cgi-bin/SMD/source/sourceResult?choice=Gene&option=Name&criteria=At4g11320) | -2.020 | Cysteine Proteinase, Putative |  |
| [At3g25830](http://genome-www4.stanford.edu/cgi-bin/SMD/source/sourceResult?choice=Gene&option=Name&criteria=At3g25830) | -1.978 | TERPENE SYNTHASE-LIKE SEQUENCE-1,8-CINEOLE | TPS-CIN |
| [At5g37990](http://genome-www4.stanford.edu/cgi-bin/SMD/source/sourceResult?choice=Gene&option=Name&criteria=At5g37990) | -1.917 | S-Adenosylmethionine-Dependent Methyltransferase |  |
| [At2g38390](http://genome-www4.stanford.edu/cgi-bin/SMD/source/sourceResult?choice=Gene&option=Name&criteria=At2g38390) | -1.890 | Peroxidase, Putative |  |
| [At1g64780](http://genome-www4.stanford.edu/cgi-bin/SMD/source/sourceResult?choice=Gene&option=Name&criteria=At1g64780) | -1.809 | AMMONIUM TRANSPORTER 1;2; Ammonium Transporter | AMT1;2 |
| [At2g13360](http://genome-www4.stanford.edu/cgi-bin/SMD/source/sourceResult?choice=Gene&option=Name&criteria=At2g13360) | -1.790 | Alanine:Glyoxylate Aminotransferase | AGT |
| [At3g27690](http://genome-www4.stanford.edu/cgi-bin/SMD/source/sourceResult?choice=Gene&option=Name&criteria=At3g27690) | -1.780 | Photosystem II Light Harvesting Complex Gene 2.3 | LHCB2.3 |
| [At3g21055](http://genome-www4.stanford.edu/cgi-bin/SMD/source/sourceResult?choice=Gene&option=Name&criteria=At3g21055) | -1.757 | PSBTN (Photosystem II Subunit T) | PSBTN |
| [At1g67870](http://genome-www4.stanford.edu/cgi-bin/SMD/source/sourceResult?choice=Gene&option=Name&criteria=At1g67870) | -1.736 | Glycine-Rich Protein |  |
| [At1g73330](http://genome-www4.stanford.edu/cgi-bin/SMD/source/sourceResult?choice=Gene&option=Name&criteria=At1g73330) | -1.678 | Arabidopsis Thaliana Drought-Repressed 4 | DR4 |
| [At2g32870](http://genome-www4.stanford.edu/cgi-bin/SMD/source/sourceResult?choice=Gene&option=Name&criteria=At2g32870) | -1.665 | Meprin And TRAF Homology Domain-Containing Protein |  |
| [At3g01500](http://genome-www4.stanford.edu/cgi-bin/SMD/source/sourceResult?choice=Gene&option=Name&criteria=At3g01500) | -1.664 | CARBONIC ANHYDRASE 1; Carbonate Dehydratase | BCA1 |
| [At5g14740](http://genome-www4.stanford.edu/cgi-bin/SMD/source/sourceResult?choice=Gene&option=Name&criteria=At5g14740) | -1.648 | Beta Carbonic Anhydrase 2 | BETA CA2 |
| [At4g12545](http://genome-www4.stanford.edu/cgi-bin/SMD/source/sourceResult?choice=Gene&option=Name&criteria=At4g12545) | -1.630 | Protease Inhibitor/Seed Storage |  |
| [At4g37580](http://genome-www4.stanford.edu/cgi-bin/SMD/source/sourceResult?choice=Gene&option=Name&criteria=At4g37580) | -1.613 | HLS1 (HOOKLESS 1); N-Acetyltransferase | COP3 |
| [At2g40170](http://genome-www4.stanford.edu/cgi-bin/SMD/source/sourceResult?choice=Gene&option=Name&criteria=At2g40170) | -1.612 | Arabidopsis Early Methionine-Labelled 6 | EM6 |
| [At5g37690](http://genome-www4.stanford.edu/cgi-bin/SMD/source/sourceResult?choice=Gene&option=Name&criteria=At5g37690) | -1.528 | GDSL-Motif Lipase/Hydrolase Family Protein |  |
| [At4g23290](http://genome-www4.stanford.edu/cgi-bin/SMD/source/sourceResult?choice=Gene&option=Name&criteria=At4g23290) | -1.521 | Protein Kinase Family Protein | CRK21 |
| [At4g12550](http://genome-www4.stanford.edu/cgi-bin/SMD/source/sourceResult?choice=Gene&option=Name&criteria=At4g12550) | -1.497 | Auxin-Induced In Root Cultures 1; Lipid Binding | AIR1 |
| [At1g19150](http://genome-www4.stanford.edu/cgi-bin/SMD/source/sourceResult?choice=Gene&option=Name&criteria=At1g19150) | -1.493 | LHCA6 (Photosystem I Light Harvesting Complex Gene 6) | LHCA2*1 |
| [At1g68110](http://genome-www4.stanford.edu/cgi-bin/SMD/source/sourceResult?choice=Gene&option=Name&criteria=At1g68110) | -1.492 | Epsin N-Terminal Homology (ENTH) Domain-Containing Protein |  |
| [At1g62510](http://genome-www4.stanford.edu/cgi-bin/SMD/source/sourceResult?choice=Gene&option=Name&criteria=At1g62510) | -1.490 | Protease Inhibitor/Seed Storage/ |  |
| [At5g17170](http://genome-www4.stanford.edu/cgi-bin/SMD/source/sourceResult?choice=Gene&option=Name&criteria=At5g17170) | -1.490 | Rubredoxin Family Protein | ENH1 |
| [At1g58290](http://genome-www4.stanford.edu/cgi-bin/SMD/source/sourceResult?choice=Gene&option=Name&criteria=At1g58290) | -1.462 | Glutamyl-Trna Reductase | HEMA1 |
| [At1g03190](http://genome-www4.stanford.edu/cgi-bin/SMD/source/sourceResult?choice=Gene&option=Name&criteria=At1g03190) | -1.461 | UVH6 (ULTRAVIOLET HYPERSENSITIVE 6) | ATXPD |
| [At3g26570](http://genome-www4.stanford.edu/cgi-bin/SMD/source/sourceResult?choice=Gene&option=Name&criteria=At3g26570) | -1.456 | PHT2;1 (Phosphate Transporter 2;1) | ORF02 |
| [At2g34620](http://genome-www4.stanford.edu/cgi-bin/SMD/source/sourceResult?choice=Gene&option=Name&criteria=At2g34620) | -1.438 | Mitochondrial Transcription Termination Factor-Related |  |
| [At4g28250](http://genome-www4.stanford.edu/cgi-bin/SMD/source/sourceResult?choice=Gene&option=Name&criteria=At4g28250) | -1.430 | Arabidopsis Thaliana Expansin B3 | EXPB3 |
| [At2g25080](http://genome-www4.stanford.edu/cgi-bin/SMD/source/sourceResult?choice=Gene&option=Name&criteria=At2g25080) | -1.420 | GLUTATHIONE PEROXIDASE 1; Glutathione Peroxidase | GPX1 |
| [At1g03130](http://genome-www4.stanford.edu/cgi-bin/SMD/source/sourceResult?choice=Gene&option=Name&criteria=At1g03130) | -1.420 | Photosystem I Subunit D-2 | PSAD-2 |
| [At3g63140](http://genome-www4.stanford.edu/cgi-bin/SMD/source/sourceResult?choice=Gene&option=Name&criteria=At3g63140) | -1.409 | RANGAP1 (RAN GTPASE ACTIVATING PROTEIN 1); | CSP41A |
| [At3g62030](http://genome-www4.stanford.edu/cgi-bin/SMD/source/sourceResult?choice=Gene&option=Name&criteria=At3g62030) | -1.385 | ROC4 (Rotamase Cyp 4); Peptidyl-Prolyl Cis-Trans Isomerase | CYP20-3 |
| [At1g70850](http://genome-www4.stanford.edu/cgi-bin/SMD/source/sourceResult?choice=Gene&option=Name&criteria=At1g70850) | -1.374 | Mlp-Like Protein 34 | MLP34 |
| [At2g05540](http://genome-www4.stanford.edu/cgi-bin/SMD/source/sourceResult?choice=Gene&option=Name&criteria=At2g05540) | -1.370 | Glycine-Rich Protein |  |
| [At5g09530](http://genome-www4.stanford.edu/cgi-bin/SMD/source/sourceResult?choice=Gene&option=Name&criteria=At5g09530) | -1.362 | Hydroxyproline-Rich Glycoprotein Family Protein | PELPK1 |
| [At4g33010](http://genome-www4.stanford.edu/cgi-bin/SMD/source/sourceResult?choice=Gene&option=Name&criteria=At4g33010) | -1.358 | Glycine Dehydrogenase (Decarboxylating), Putative | GLDP1 |
| [At3g59400](http://genome-www4.stanford.edu/cgi-bin/SMD/source/sourceResult?choice=Gene&option=Name&criteria=At3g59400) | -1.358 | Genomes Uncoupled 4 | GUN4 |
| [At1g65230](http://genome-www4.stanford.edu/cgi-bin/SMD/source/sourceResult?choice=Gene&option=Name&criteria=At1g65230) | -1.344 | Similar To Mtrdraft_AC148171g2v1 |  |
| [At1g45201](http://genome-www4.stanford.edu/cgi-bin/SMD/source/sourceResult?choice=Gene&option=Name&criteria=At1g45201) | -1.343 | Triacylglycerol Lipase | TLL1 |
| [At2g40100](http://genome-www4.stanford.edu/cgi-bin/SMD/source/sourceResult?choice=Gene&option=Name&criteria=At2g40100) | -1.342 | LIGHT HARVESTING COMPLEX PSII; Chlorophyll Binding | LHCB4.3 |
| [At5g45820](http://genome-www4.stanford.edu/cgi-bin/SMD/source/sourceResult?choice=Gene&option=Name&criteria=At5g45820) | -1.331 | CBL-INTERACTING PROTEIN KINASE 20; Kinase | CIPK20 |
| [At1g52870](http://genome-www4.stanford.edu/cgi-bin/SMD/source/sourceResult?choice=Gene&option=Name&criteria=At1g52870) | -1.325 | Peroxisomal Membrane Protein-Related |  |
| [At3g61870](http://genome-www4.stanford.edu/cgi-bin/SMD/source/sourceResult?choice=Gene&option=Name&criteria=At3g61870) | -1.315 | Similar To Conserved Hypothetical Protein [Medicago Truncatula] |  |
| [At1g16720](http://genome-www4.stanford.edu/cgi-bin/SMD/source/sourceResult?choice=Gene&option=Name&criteria=At1g16720) | -1.313 | Oxidoreductase/ Transcriptional Repressor | HCF173 |
| [At5g35170](http://genome-www4.stanford.edu/cgi-bin/SMD/source/sourceResult?choice=Gene&option=Name&criteria=At5g35170) | -1.309 | Nucleotide Kinase |  |
| [At1g75460](http://genome-www4.stanford.edu/cgi-bin/SMD/source/sourceResult?choice=Gene&option=Name&criteria=At1g75460) | -1.305 | ATP-Dependent Protease La (LON) Domain-Containing Protein |  |
| [At5g20250](http://genome-www4.stanford.edu/cgi-bin/SMD/source/sourceResult?choice=Gene&option=Name&criteria=At5g20250) | -1.305 | DARK INDUCIBLE 10; Hydrolase, | DIN10 |
| [At2g39730](http://genome-www4.stanford.edu/cgi-bin/SMD/source/sourceResult?choice=Gene&option=Name&criteria=At2g39730) | -1.301 | Rubisco Activase | RCA |
| [At3g55850](http://genome-www4.stanford.edu/cgi-bin/SMD/source/sourceResult?choice=Gene&option=Name&criteria=At3g55850) | -1.300 | LAF3/LAF3 ISF1/LAF3 ISF2 (LONG AFTER FAR-RED 3); Hydrolase | LAF3 |
| [At5g01530](http://genome-www4.stanford.edu/cgi-bin/SMD/source/sourceResult?choice=Gene&option=Name&criteria=At5g01530) | -1.285 | Chlorophyll A-B Binding Protein CP29 (LHCB4) | LHCB4.1 |
| [At3g47070](http://genome-www4.stanford.edu/cgi-bin/SMD/source/sourceResult?choice=Gene&option=Name&criteria=At3g47070) | -1.273 | Similar To GB:CAD45559.1 |  |
| [At4g32260](http://genome-www4.stanford.edu/cgi-bin/SMD/source/sourceResult?choice=Gene&option=Name&criteria=At4g32260) | -1.243 | ATP Synthase Family | PDE334 |
| [At4g27700](http://genome-www4.stanford.edu/cgi-bin/SMD/source/sourceResult?choice=Gene&option=Name&criteria=At4g27700) | -1.241 | Rhodanese-Like Domain-Containing Protein |  |
| [At1g76080](http://genome-www4.stanford.edu/cgi-bin/SMD/source/sourceResult?choice=Gene&option=Name&criteria=At1g76080) | -1.212 | ATCDSP32/CDSP32 | CDSP32 |
| [At3g52720](http://genome-www4.stanford.edu/cgi-bin/SMD/source/sourceResult?choice=Gene&option=Name&criteria=At3g52720) | -1.211 | Carbonic Anhydrase Family Protein | ACA1 |
| [At4g39010](http://genome-www4.stanford.edu/cgi-bin/SMD/source/sourceResult?choice=Gene&option=Name&criteria=At4g39010) | -1.184 | Glycosyl Hydrolase Family 9 Protein | GH9B18 |
| [At5g47040](http://genome-www4.stanford.edu/cgi-bin/SMD/source/sourceResult?choice=Gene&option=Name&criteria=At5g47040) | -1.167 | Lon Protease Homolog 1, Mitochondrial (LON) | LON2 |
| [At2g33850](http://genome-www4.stanford.edu/cgi-bin/SMD/source/sourceResult?choice=Gene&option=Name&criteria=At2g33850) | -1.163 | Similar To TAIR:AT1G28400.1 |  |
| [At3g46780](http://genome-www4.stanford.edu/cgi-bin/SMD/source/sourceResult?choice=Gene&option=Name&criteria=At3g46780) | -1.159 | Plastid Transcriptionally Active18 | PTAC16 |
| [At2g26080](http://genome-www4.stanford.edu/cgi-bin/SMD/source/sourceResult?choice=Gene&option=Name&criteria=At2g26080) | -1.158 | Glycine Dehydrogenase (Decarboxylating), | GLDP2 |
| [At5g65730](http://genome-www4.stanford.edu/cgi-bin/SMD/source/sourceResult?choice=Gene&option=Name&criteria=At5g65730) | -1.149 | Xyloglucan:Xyloglucosyl Transferase, | XTH6 |
| [At1g51400](http://genome-www4.stanford.edu/cgi-bin/SMD/source/sourceResult?choice=Gene&option=Name&criteria=At1g51400) | -1.137 | Photosystem II 5 Kd Protein |  |
| [At5g21222](http://genome-www4.stanford.edu/cgi-bin/SMD/source/sourceResult?choice=Gene&option=Name&criteria=At5g21222) | -1.131 | Protein Kinase Family Protein |  |
| [At1g77490](http://genome-www4.stanford.edu/cgi-bin/SMD/source/sourceResult?choice=Gene&option=Name&criteria=At1g77490) | -1.130 | L-Ascorbate Peroxidase | TAPX |
| [At2g05880](http://genome-www4.stanford.edu/cgi-bin/SMD/source/sourceResult?choice=Gene&option=Name&criteria=At2g05880) | -1.127 | Replication Protein-Related |  |
| [At5g51010](http://genome-www4.stanford.edu/cgi-bin/SMD/source/sourceResult?choice=Gene&option=Name&criteria=At5g51010) | -1.124 | Rubredoxin Family Protein |  |
| [At1g15380](http://genome-www4.stanford.edu/cgi-bin/SMD/source/sourceResult?choice=Gene&option=Name&criteria=At1g15380) | -1.122 | Lactoylglutathione Lyase | GLYI4 |
| [At1g68010](http://genome-www4.stanford.edu/cgi-bin/SMD/source/sourceResult?choice=Gene&option=Name&criteria=At1g68010) | -1.120 | HPR (Hydroxypyruvate Reductase); NAD Binding / Cofactor Binding | HPR1 |
| [At5g17320](http://genome-www4.stanford.edu/cgi-bin/SMD/source/sourceResult?choice=Gene&option=Name&criteria=At5g17320) | -1.071 | Homeobox-Leucine Zipper Family Protein | HDG9 |
| [At4g12830](http://genome-www4.stanford.edu/cgi-bin/SMD/source/sourceResult?choice=Gene&option=Name&criteria=At4g12830) | -1.067 | Hydrolase, Alpha/Beta Fold Family Protein |  |
| [At3g14420](http://genome-www4.stanford.edu/cgi-bin/SMD/source/sourceResult?choice=Gene&option=Name&criteria=At3g14420) | -1.067 | (S)-2-Hydroxy-Acid Oxidase, Peroxisomal, Putative |  |
| [At4g09650](http://genome-www4.stanford.edu/cgi-bin/SMD/source/sourceResult?choice=Gene&option=Name&criteria=At4g09650) | -1.060 | ATP Synthase Delta Chain, Chloroplast, Putative | ATPD |
| [At1g43040](http://genome-www4.stanford.edu/cgi-bin/SMD/source/sourceResult?choice=Gene&option=Name&criteria=At1g43040) | -1.027 | Auxin-Responsive Protein, Putative |  |

**Supplementary Table SIII. Gene Ontology of the Biological Processes of the genes differentially regulated in *COPT1^OE^* seedlings.** NG, number of annotated genes in the input list; TNG, total number of genes in the input list; NGR, number of annotated genes in the reference list; TNGR, total number of genes in the reference list; Hyp*, corrected hypergeometric p-Value.

|  | **Gene Ontology (Biological Process)** | **NG/TNG** | **NGR/TNGR** | **Hyp_c** |  |  |  |
| --- | --- | --- | --- | --- | --- | --- | --- |
| **Down-regulated** | photosynthesis | 0.0396 | 0.0011 | 0.0002 |  |  |  |
|  | response to light stimulus | 0.0495 | 0.0022 | 0.0002 |  |  |  |
|  | glycine decarboxylation via glycine cleavage system | 0.0198 | 0.0001 | 0.0007 |  |  |  |
|  | iron ion transport | 0.0198 | 0.0002 | 0.0049 |  |  |  |
|  | zinc ion transport | 0.0198 | 0.0003 | 0.0050 |  |  |  |
|  | cellular iron ion homeostasis | 0.0198 | 0.0004 | 0.0077 |  |  |  |
|  | indole glucosinolate metabolic process | 0.0198 | 0.0004 | 0.0079 |  |  |  |
|  | unidimensional cell growth | 0.0297 | 0.0016 | 0.0081 |  |  |  |
|  | protein-chromophore linkage | 0.0198 | 0.0006 | 0.0130 |  |  |  |
|  | chlorophyll biosynthetic process | 0.0198 | 0.0006 | 0.0130 |  |  |  |
|  | phosphate ion transport | 0.0099 | 0.0001 | 0.0316 |  |  |  |
|  | phosphate ion transmembrane transport | 0.0099 | 0.0001 | 0.0316 |  |  |  |
|  | plastid transcription | 0.0099 | 0.0001 | 0.0316 |  |  |  |
|  | syncytium formation | 0.0099 | 0.0001 | 0.0316 |  |  |  |
|  | oxidation-reduction process | 0.0495 | 0.0110 | 0.0374 |  |  |  |
|  | chloroplast-nucleus signaling pathway | 0.0099 | 0.0001 | 0.0395 |  |  |  |
|  | nitrile biosynthetic process | 0.0099 | 0.0001 | 0.0395 |  |  |  |
|  | phosphoenolpyruvate transport | 0.0099 | 0.0001 | 0.0395 |  |  |  |
|  | glyoxylate cycle | 0.0099 | 0.0002 | 0.0412 |  |  |  |
|  | protein targeting to peroxisome | 0.0099 | 0.0002 | 0.0412 |  |  |  |
|  | lateral root morphogenesis | 0.0099 | 0.0002 | 0.0412 |  |  |  |
|  | galactose catabolic process | 0.0099 | 0.0002 | 0.0412 |  |  |  |
|  | protein processing | 0.0099 | 0.0002 | 0.0412 |  |  |  |
|  | cadmium ion transport | 0.0099 | 0.0002 | 0.0412 |  |  |  |
|  | iron ion homeostasis | 0.0099 | 0.0002 | 0.0412 |  |  |  |
|  | photosynthetic electron transport in photosystem I | 0.0099 | 0.0002 | 0.0412 |  |  |  |
|  | heme biosynthetic process | 0.0099 | 0.0002 | 0.0448 |  |  |  |
|  | protein targeting to chloroplast | 0.0099 | 0.0002 | 0.0448 |  |  |  |
|  | response to abscisic acid | 0.0198 | 0.0021 | 0.0469 |  |  |  |
|  | galactose catabolic process via UDP-galactose | 0.0099 | 0.0002 | 0.0471 |  |  |  |
|  | photosynthesis, light harvesting | 0.0099 | 0.0002 | 0.0471 |  |  |  |
|  | response to iron ion | 0.0099 | 0.0002 | 0.0471 |  |  |  |
|  | manganese ion transport | 0.0099 | 0.0002 | 0.0471 |  |  |  |
|  | positive regulation of catalytic activity | 0.0099 | 0.0002 | 0.0471 |  |  |  |
|  | phosphoglycerate transmembrane transport | 0.0099 | 0.0002 | 0.0471 |  |  |  |
|  | response to freezing | 0.0099 | 0.0001 | 0.0473 |  |  |  |
|  | photorespiration | 0.0099 | 0.0001 | 0.0473 |  |  |  |
|  | regulation of stomatal movement | 0.0099 | 0.0003 | 0.0480 |  |  |  |
|  | porphyrin-containing compound biosynthetic process | 0.0099 | 0.0003 | 0.0480 |  |  |  |
|  | response to cold | 0.0198 | 0.0026 | 0.0498 |  |  |  |
| **Up-regulated** | defense response | 0.0211 | 0.0047 | 0.0051 |  |  |  |
|  | cellular oxidant detoxification | 0.0063 | 0.0003 | 0.0081 |  |  |  |
|  | cellular response to sulfur starvation | 0.0042 | 0.0001 | 0.0091 |  |  |  |
|  | aging | 0.0084 | 0.0010 | 0.0355 |  |  |  |
|  | response to bacterium | 0.0084 | 0.0011 | 0.0454 |  |  |  |

**Supplementary Table SIV. Gene Ontology of the Molecular Function of the genes differentially regulated in *COPT1^OE^* seedlings.** NG, number of annotated genes in the input list; TNG, total number of genes in the input list; NGR, number of annotated genes in the reference list; TNGR, total number of genes in the reference list; Hyp*, corrected hypergeometric p-Value.

|  |  |  |  |  |
| --- | --- | --- | --- | --- |
|  | **Gene Ontology (Molecular Function)** | **NG/TNG** | **NGR/TNGR** | **Hyp_c** |
| **Down-regulated** | enzyme regulator activity | 0.0198 | 0.0002 | 0.0186 |
|  | chlorophyll binding | 0.0198 | 0.0008 | 0.0201 |
|  | low-affinity phosphate transmembrane transporter activity | 0.0099 | 0.0000 | 0.0205 |
|  | glycerate dehydrogenase activity | 0.0099 | 0.0000 | 0.0205 |
|  | hydroxypyruvate reductase activity | 0.0099 | 0.0000 | 0.0205 |
|  | ribulose-1,5-bisphosphate carboxylase/oxygenase activator activity | 0.0099 | 0.0000 | 0.0205 |
|  | phosphoenolpyruvate:phosphate antiporter activity | 0.0099 | 0.0000 | 0.0205 |
|  | glycolate oxidase activity | 0.0099 | 0.0000 | 0.0205 |
|  | tetrapyrrole binding | 0.0099 | 0.0000 | 0.0205 |
|  | glycine dehydrogenase (decarboxylating) activity | 0.0099 | 0.0000 | 0.0205 |
|  | glycine binding | 0.0099 | 0.0000 | 0.0205 |
|  | isocitrate lyase activity | 0.0099 | 0.0000 | 0.0205 |
|  | carbohydrate kinase activity | 0.0099 | 0.0001 | 0.0281 |
|  | efflux transmembrane transporter activity | 0.0099 | 0.0001 | 0.0281 |
|  | transferase activity, transferring acyl groups other than amino-acyl groups | 0.0099 | 0.0001 | 0.0281 |
|  | phospholipid-hydroperoxide glutathione peroxidase activity | 0.0099 | 0.0001 | 0.0281 |
|  | carbonate dehydratase activity | 0.0099 | 0.0001 | 0.0293 |
|  | glutamyl-tRNA reductase activity | 0.0099 | 0.0001 | 0.0293 |
|  | calcium channel activity | 0.0099 | 0.0001 | 0.0293 |
|  | ferroxidase activity | 0.0099 | 0.0001 | 0.0293 |
|  | glyoxylate reductase (NADP) activity | 0.0099 | 0.0001 | 0.0293 |
|  | iron ion transmembrane transporter activity | 0.0099 | 0.0001 | 0.0293 |
|  | enzyme binding | 0.0099 | 0.0001 | 0.0293 |
|  | iron ion binding | 0.0099 | 0.0001 | 0.0374 |
|  | serine-type peptidase activity | 0.0099 | 0.0002 | 0.0433 |
|  | UDP-glucose 4-epimerase activity | 0.0099 | 0.0002 | 0.0433 |
|  | N-acetyltransferase activity | 0.0099 | 0.0002 | 0.0433 |
|  | peptidyl-prolyl cis-trans isomerase activity | 0.0099 | 0.0002 | 0.0433 |
|  | phosphoglycerate transmembrane transporter activity | 0.0099 | 0.0002 | 0.0433 |
|  | triose-phosphate transmembrane transporter activity | 0.0099 | 0.0002 | 0.0433 |
|  | inorganic phosphate transmembrane transporter activity | 0.0099 | 0.0001 | 0.0448 |
| **Up-regulated** | glutathione transferase activity | 0.0105 | 0.0008 | 0.0053 |
|  | nitrilase activity | 0.0042 | 0.0001 | 0.0302 |
|  | indole-3-acetonitrile nitrile hydratase activity | 0.0042 | 0.0001 | 0.0302 |
|  | indole-3-acetonitrile nitrilase activity | 0.0042 | 0.0001 | 0.0302 |

**Supplementary Table SV. Gene Ontology of the Cellular Compartment of the genes differentially regulated in *COPT1^OE^* seedlings.** NG, number of annotated genes in the input list; TNG, total number of genes in the input list; NGR, number of annotated genes in the reference list; TNGR, total number of genes in the reference list; Hyp*, corrected hypergeometric p-Value.

|  | **Gene Ontology (Cellular Compartment)** | **NG/TNG** | **NGR/TNGR** | **Hyp_c** |
| --- | --- | --- | --- | --- |
| **Down-regulated** | chloroplast envelope | 0.1485 | 0.0065 | 0.000000001 |
|  | chloroplast | 0.2772 | 0.0537 | 0.000000057 |
|  | photosystem II | 0.0297 | 0.0008 | 0.000497440 |
|  | chloroplast thylakoid membrane | 0.0990 | 0.0059 | 0.000549245 |
|  | plastoglobule | 0.0198 | 0.0002 | 0.000754412 |
|  | chloroplast stroma | 0.0396 | 0.0032 | 0.001574810 |
|  | light-harvesting complex | 0.0198 | 0.0004 | 0.002902050 |
|  | chloroplast membrane | 0.0297 | 0.0022 | 0.004784660 |
|  | integral component of membrane | 0.1287 | 0.0491 | 0.005037790 |
|  | chloroplast inner membrane | 0.0198 | 0.0008 | 0.007858580 |
|  | photosystem I | 0.0198 | 0.0008 | 0.007858580 |
|  | cytosol | 0.0495 | 0.0107 | 0.012148000 |
|  | early endosome | 0.0099 | 0.0001 | 0.013605900 |
|  | peroxisomal matrix | 0.0099 | 0.0001 | 0.013605900 |
|  | cytoplasm | 0.0990 | 0.0440 | 0.025392900 |
|  | thylakoid | 0.0198 | 0.0016 | 0.025450600 |
|  | peroxisome | 0.0198 | 0.0017 | 0.026508500 |
|  | proton-transporting ATP synthase complex, catalytic core F(1) | 0.0099 | 0.0001 | 0.026734200 |
|  | perinuclear region of cytoplasm | 0.0099 | 0.0002 | 0.030431700 |
|  | chloroplast photosystem II | 0.0099 | 0.0002 | 0.038528800 |

**Supplementary Table SVI**. **Primers used for the RT-qPCR analysis**. The primers name and their sequence (5´to 3´) are indicated.

| **primer name** | **primer sequence (5’ to 3’)** |
| --- | --- |
| UBQ10 F1  UBQ10 R1 | TAATCCCTGATGAATAAGTGTTCTAC  AAAACGAAGCGATGATAAAGAAG |
| IRT1 F qPCR  IRT1 R qPCR | CCCCGCAAATGATGTTACCTT  GGTATCGCAAGAGCTGTGCAT |
| FER1 qRT F  FER1 qRT R | CACCCAGCTAAGGATGATCG  TCAGAAGCATCTGGTCGAAA |
| FER3 qPCR-F  FER3 qPCR-R | AGAGTGTGTTTCTGAACGAAC  CCAAACTGCGAGATTACAGC |
| At-NEET F qPCR  At-NEET R qPCR | AAGCAGCAGAGAATGGTGGT  TGCTTCACATGACTCCCATC |
| FIT F qPCR  FIT R qPCR | TTTTCGCGGTATCAATCCTC  GGTATGTGTCCGGAGAAGGA |
| bHLH39 F qPCR  bHLH39 R qPCR | CAGAGCTGCAAGAGCAAGTG  ACCAAGCCTAGTCGCAGAAA |
| bHLH100 F qPCR  bHLH100 R qPCR | AAACCGACGACGTATCCAAC  GATTGGTGGGAGGAGACAA |
| FEP2 QPCR F  FEP2 QPCR R | CCTCATCACACTTCTTCACTCGGTG  GGAGACAATCACGCAGCAGGAGC |
| FEP3 QPCR F  FEP3 QPCR R | GTGGCTTTCTCTTTACACCTCTTCATTGC  CGCAGCAGGAGCATAATCATAGCC |
| BRUTUS qPCR F  BRUTUS qPCR R | GCTCTGGCACAAGTCAATCA  CGTTCATCAAATGCCGATAA |
| LHCB2.3 qPCR F  LHCB2.3 qPCR R | GGCGTGTCAAGTTGTGCTAA  GAACATAGCAAGACGACCGT |
| GUN4 F qPCR  GUN4 R qPCR | TTAGGACACTTACCGCTCAC  GCTCGTCTTCTGTTTCTCCA |
| CRD1 F QPCR  CRD1 R QPCR | GCGAGTGCTACCTTCTCCTT  GCGAGAATGGTGAGATGGGT |
| SIG1 F qPCR  SIG1 R qPCR | CGGTGTCGAGTGAGTATCCG  CAAGCGCACAACTTCCACAT |
| AOX1D F qPCR  AOX1D R qPCR | CCATGCGATGTTGCTAGAGA  AAGCCAGGAAATATGCGTTG |
| LSU1 qRT F  LSU1 qRT R | AGGCGGAAGAGCAACTCTG  TTGATCCATGAGGAAGAGCA |
| UPB1 qRT F  UPB1 qRT R | AAGACCTTGTCGCGCAATAC  TTTCGGTCAAAACCTGAACC |
| SPL7 q-F  SPL7 q-R | CAGGCAGACTGTTCACCAGA  AGTTTGACGGGACCTGAATG |
| COPT2 qRT-F  COPT2 qRT-R | CCTTTCGTATTTGGTGATGCT  AAACACCTGCGTTAAAGGAC |
| FSD1 qRT-F  FSD1 qRT-R | ACCGAAGACCAGATTACATA  TGGCACTTACAGCTTCCCAA |
| CSD2 qRT-F  CSD2 qRT-R | GTCCTACAACTGTGAAT  TCCATGAGGCCCTGGAGT |
| BHLH038 QRT F  BHLH038 QRT R | AGAGCTGCAACAGCAAGTGA  ACCAAGCCTAGTGGCAGAAA |
| BHLH101 F qPCR  BHLH101 R qPCR | TTGCTGTCCAGTTGCTACG  GGCGTAATCCCAAGAGACATA |
| BHLH115 qPCR f  BHLH115 qPCR r | ACGAGCTGCGTGATGAGAAA  AGGAGGCATGAATTGCCACA |
| ILR3 qPCR f  ILR3 qPCR r | GCCCCACCTATGATGCCTAC  CACATGGCAACTCCTGGGTA |
| PER39 qPCR-F  PER39 qPCR-R | TTTCATGACTGCTTCGTTCG  TGATATCAGCGCAAGAGACG |
| PER40 qPCR-F  PER40 qPCR-R | TCTACTCGTGGGTGGAAACC  CAATCACTTCGAACCCTCGT |

**
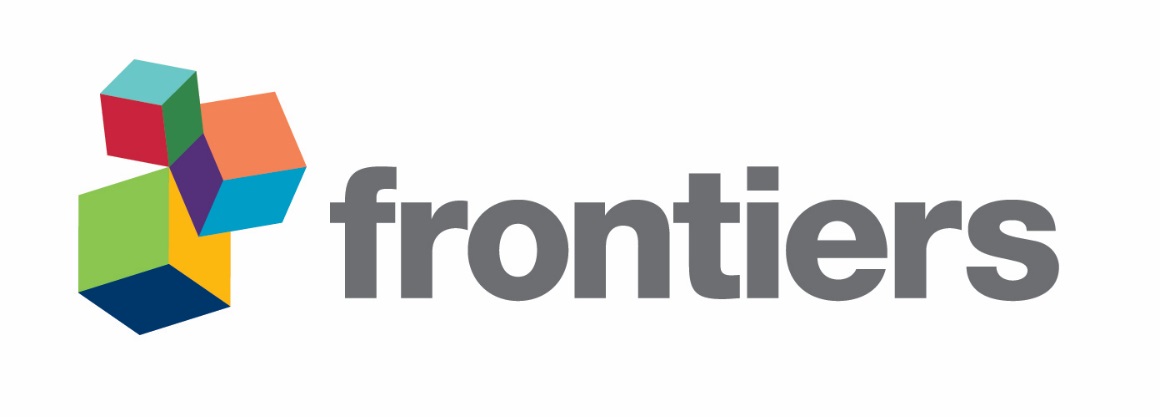
**
